# Investigation of Antioxidant, In Silico and In Vivo Antiulcer Activities of New Natural Xanthenone and Antracenone Isolated from *Tricholaena teneriffae* L. Roots

**DOI:** 10.3390/molecules31111850

**Published:** 2026-05-28

**Authors:** Henda Keskes, Siwar Soltani, Khaled Hamden, Anthony Abou Dib, Jean-Hugues Renault, Musafau Sanni, Abdel Halim Harrath, Noureddine Allouche, Hichem Ben Salah

**Affiliations:** 1 Laboratory of Organic Chemistry LR17ES08, Natural Substances Team, Faculty of Sciences of Sfax, University of Sfax, Sfax 3000, Tunisia; hendakeskes@yahoo.fr (H.K.); siwarsoltani90@gmail.com (S.S.); hichembensalah9@gmail.com (H.B.S.); 2 Laboratory of Bioresources, Integrative Biology and Exploiting, Higher Institute of Biotechnology of Monastir, University of Monastir, Monastir 5000, Tunisia; khaledhamden@yahoo.fr; 3 Reims Institute of Molecular Chemistry (ICMR), UMR CNRS 7312, University of Reims Champagne-Ardenne, 51687 Reims, Francejean-huguesrenault@univ-reins.fr (J.-H.R.); 4 Department of Zoology, College of Science, King Saud University, Riyadh 11451, Saudi Arabiahharrath@ksu.edu.sa (A.H.H.)

**Keywords:** *Tricholaena teneriffae* L., antioxidant, gastroprotective effect, molecular docking

## Abstract

This study aims to investigate the antioxidant, anti-inflammatory, and anti-ulcer properties of two newly isolated natural compounds (**1**–**2**) from the hydroalcoholic root extract of *Tricholaena teneriffae* L. Through bioassay-guided fractionation, two new natural compounds, xantenone and anthracenone, were isolated and characterized using 1D/2D NMR and mass spectrometry. While 6-hydroxy-3-methoxy-7H-benzo[*de*]anthracen-7-one (**HK2**) showed significant antioxidant activity, 11-hydroxy-12H-benzo[a]xanthen-12-one (**HK1**) exhibited only weak activity. However, both compounds demonstrated a gastroprotective effect in vivo, similar to that of omeprazole. In silico screening revealed that both molecules bind stably to the H^+^/K^+^-ATPase proton pump, suggesting a potential mechanism for inhibiting gastric acid secretion. In the ethanol-induced ulcer model, treatment with **HK1** and **HK2** significantly reduced inflammation, oxidative stress, gastric hypersecretion, and ulcer area, with **HK2** showing greater efficacy. These results suggest that the active fraction of *T. teneriffae* root has anti-ulcer properties beyond its antioxidant effects, making it a promising natural source of gastroprotective agents.

## 1. Introduction

Peptic ulcer disease is a prevalent gastrointestinal disorder responsible for a substantial number of deaths each year [1,2,3]. It results from the erosion of the gastric or duodenal mucosa [4], manifesting as persistent abdominal pain often accompanied by digestive discomfort [5]. Although multiple factors, such as alcohol consumption, tobacco use, prolonged intake of anti-inflammatory drugs [6], and specific cancer therapies, increase the risk of ulcer development, current pharmacological treatments are frequently associated with adverse effects [7]. This has intensified research interest in medicinal plants as therapeutic alternatives due to their richness in bioactive metabolites [8].

Within this framework, the Poaceae family has emerged as a promising source of phytochemicals with significant biological potential, especially phenolic compounds such as xanthones—oxygen-containing heterocycles with a γ-pyrone moiety fused to two benzene rings [9]. *Tricholaena teneriffae* L. (Poaceae) is a wild grass that is widely distributed across arid and semi-arid environments. It is recognized for its strong resilience to various environmental stresses, including salinity and drought. Despite its ecological significance, the phytochemical profile of this species remains largely understudied. To date, only one study has examined the chemical composition of its aerial parts, which revealed a high concentration of phenolic acids in alcoholic extracts. The methanolic extract was found to possess the strongest antioxidant and antibacterial activities among the tested fractions [10].

Notably, the phytochemical profile of *T. teneriffae* roots has not yet been thoroughly explored, representing a significant gap in the current knowledge of this species. To address this, the present study describes the isolation and characterization of two novel natural products from the hydroalcoholic root extract: a benzo[*a*]xanthenone derivative (**HK1**) and a benzo[*de*]anthracenone derivative (**HK2**). While their basic chemical skeletons are documented in the literature, these specific derivatives are reported here as new natural compounds for the first time. Their identification provides not only new chemotaxonomic insights into the *Tricholaena* genus but also highlights the unique biosynthetic potential of its roots.

The chemical structures were elucidated through rigorous spectroscopic analyses, including 2D NMR correlations (HSQC and HMBC) to ensure precise positional assignment of substituents. This study also evaluates the total phenolic and flavonoid content, as well as the radical-scavenging and reducing capacities of the extract and the two isolates. Furthermore, an in vivo investigation was conducted on albino Wistar rats to assess the anti-ulcer and anti-inflammatory activities. These assessments serve as a preliminary screening of the pharmacological potential of these metabolites. Finally, molecular docking analysis targeting the H^+^/K^+^-ATPase enzyme was performed to explore potential binding modes and provide a theoretical basis for the observed gastroprotective effects.

## 2. Results and Discussion

70% ethanol (70% ethanol and 30% water) was chosen for its ability to generate non-selective extracts via solid-liquid extraction from the dry powder of *T. teneriffae* and for its bio-based nature in line with the principles of eco-extraction [11].

The structures of 11-hydroxy-12H-benzo[*a*]xanthen-12-one and 6-hydroxy-3-methoxy-7H-benzo[*de*]anthracen-7-one were confirmed using HR-ESI-MS, IR, 1D and 2D homo- and heteronuclear NMR analyses.

### 2.1. Characterization of 11-Hydroxy-12H-benzo[a]xanthen-12-one (HK1)

Compound **1** (**HK1**) was obtained as a yellow powder (30 mg). Its molecular formula was established as C_17_H_10_O_3_ based on high-resolution electrospray ionization mass spectrometry (HRESIMS, Figure S1), which exhibited a pseudo-molecular ion peak at *m*/*z* 263.0729 [M + H]^+^, (calculated for C_17_H_11_O_3_, 263.0708), indicating for thirteen degrees of unsaturation. The UV–Vis spectrum of **HK1** in CH_2_Cl_2_ (Figure S2) showed absorption maxima at 238, 316, and 383 nm, consistent with its extended conjugated system. Together with the IR bands at 3228 (O–H) and 1640 cm^−1^ (conjugated C=O) (Figure S3), these data fully support the functional group assignments for the benzo[a]xanthen-12-one skeleton.

**Figure 1:**
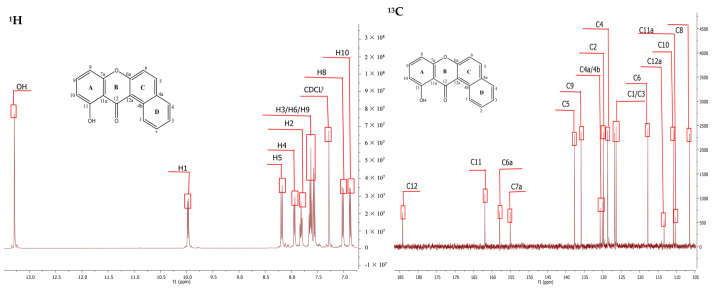
^1^H and ^13^C NMR spectra of **HK1** in CDCL_3_.

The ^1^H NMR spectrum (Figure 1 and Figure S4) revealed a sharp singlet at *δ_H_* 13.29 ppm, attributed to a peri-hydroxyl proton involved in strong intramolecular hydrogen bonding with the adjacent carbonyl (C-12, *δ_C_* 184.23). Combined analysis of ^13^C NMR, DEPT, and HSQC spectra (Figures S5–S7) revealed 17 carbon signals, including one carbonyl at *δ*_C_ 184.23, nine aromatic methines, and seven quaternary carbons. Among these, one oxygenated carbon at *δ*_C_ 161.93 (C-11) was also identified (Table 1).

**Figure 2:**
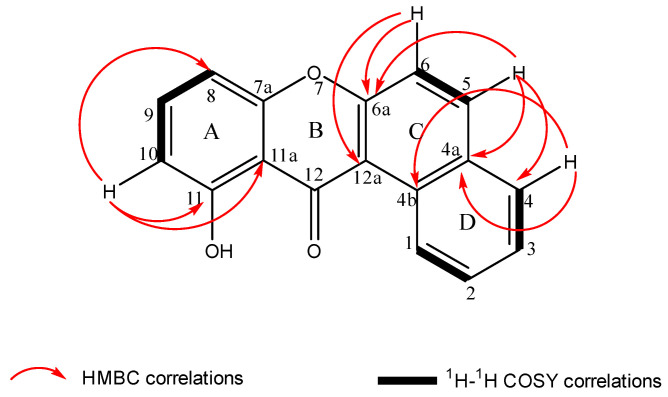
Key HMBC and ^1^H-^1^H COSY correlations for compound **HK1**.

Further examination of HMBC and COSY (Figures S8 and S9) correlations definitively confirmed the tetracyclic structure (Figure 1). While the ^13^C NMR data for **HK1** showed close agreement with literature values for synthetic 11-amino-12H-benzo[a]xanthen-12-one [12] regarding rings B, C, and D, significant differences were noted in ring A (Table 1). Specifically, the absence of an amine signal (*δ*_H_ 7.70, br s, NH_2_) and the presence of the chelated hydroxyl signal at *δ*_H_ 13.29 ppm confirmed the substitution. ^1^H–^1^H COSY analysis revealed a spin system between H-8 (*δ*_H_ 7.02, 1H, d, ^3^*J* = 8 Hz), H-9 (*δ*_H_ 7.62, 1H, d, ^3^*J* = 8.4 Hz), and H-10 (*δ*_H_ 6.88, 1H, m), indicating a 1,2,3-trisubstituted aromatic ring A (Figure 1). This was further supported by HMBC correlations of H-10 (*δ*_H_ 6.88) with an oxygenated carbon (*δ*_C_ 161.93), C-11a (*δ*_C_ 110.37), and C-8 (*δ*_C_ 106.50), which confirmed the location of the hydroxyl group at C-11 in ring A (Figure 2). These findings confirm the discovery of a novel natural derivative compound, 11-hydroxy-12H-benzo[*a*]xanthen-12-one, which was identified for the first time in the hydroalcoholic extract of *T. teneriffae*.

### 2.2. Characterization of 6-Hydroxy-3-methoxy-7H-benzo[de]anthracen-7-one (HK2)

Compound 2 (**HK2**) was obtained as a yellow powder (52 mg). Its molecular formula was established as C_18_H_12_O_3_ by HRESIMS (Figure S10), which showed a pseudo-molecular ion peak at *m*/*z* 277.0869 [M + H]^+^ (calculated for C_18_H_13_O_3_, 277.0865). This indicates the presence of thirteen degrees of unsaturation. The UV–Vis spectrum in CH_2_Cl_2_ (Figure S11) exhibited absorption maxima at 234, 260, 302, and 325 nm, a pattern characteristic of a highly conjugated polycyclic aromatic system. The IR spectrum (Figure S12) showed a characteristic absorption band at 1610 cm^−1^ for the chelated conjugated carbonyl group (C=O). The methoxy group was identified by its C-H stretching at 2917 cm^−1^ and the strong C-O-C signal at 1233 cm^−1^. The broad band at 3178 cm^−1^ confirmed the presence of the hydroxyl group involved in intramolecular hydrogen bonding.

**Figure 3:**
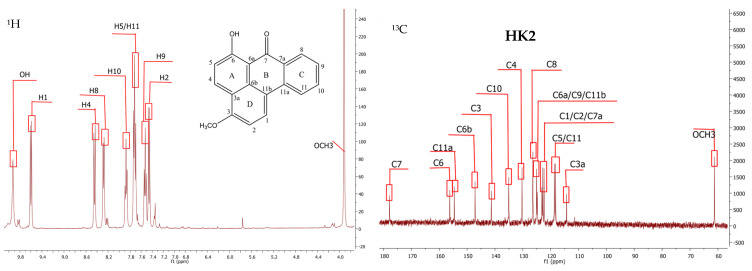
^1^H and ^13^C NMR spectra of **HK2** in DMSO-d_6_.

Combined analysis of the ^13^C NMR, DEPT, and HSQC spectra (Figure 3 and Figures S13–S15) revealed 18 carbon signals, including a conjugated carbonyl at *δ_C_* 177.89 (C-7), a methoxy group at *δ_C_* 61.24, eight aromatic methines, and nine quaternary carbons (Table 2). The ^1^H NMR spectrum of **HK2** (Figure S16) showed a methoxy group at *δ_H_* 3.92 (3H, s) and a chelated phenolic hydroxyl group at *δ_H_* 9.93 (1H, s). A highly deshielded doublet was observed at *δ_H_* 9.60 (*J* = 9.2 Hz, H-1), which is characteristic of a proton in a benzanhrone skeleton due to the magnetic anisotropy of the neighboring carbonyl group. The aromatic region further revealed three distinct doublets at *δ_H_* 8.45 (*J* = 9.2 Hz, H-4), 7.46 (*J* = 9.2 Hz, H-2), and 8.28 (*J* = 8.4 Hz, H-8). The signal at *δ_H_* 7.53 (m) was assigned to H-9, while the overlapping multiplet at *δ_H_* 7.70–7.73 (2H) was attributed to H-5 and H-11, consistent with the proposed substitution pattern.

**Figure 4:**
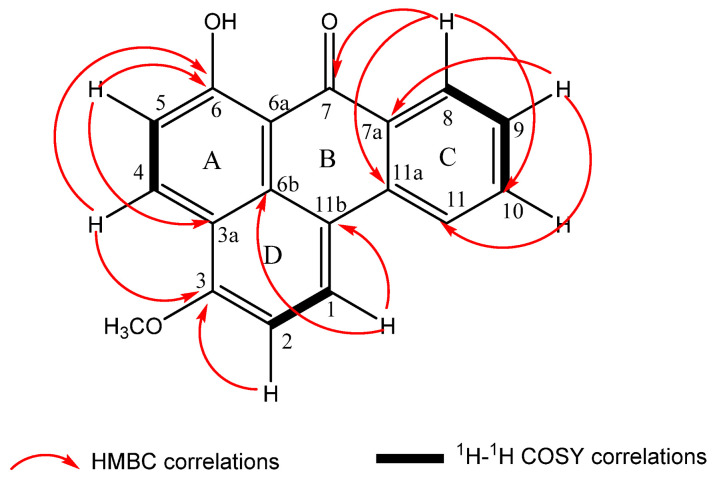
Key HMBC and ^1^H-^1^H COSY correlations for compound **HK2**.

In addition, ^1^H–^1^H COSY analysis (Figure 4 and Figure S17) showed that the proton H-5 (*δ_H_* 7.70) correlated with H-4 (*δ_H_* 8.45). In the HMBC spectrum (Figure 4 and Figure S18), both H-4 and H-5 showed clear correlations with an oxygenated aromatic carbon at *δ_C_* 156.23 (C-6), confirming the presence of a hydroxyl group at this position on ring A (Figure 4). Further, the protons H-1 (*δ_H_* 9.60) and H-2 (*δ_H_* 7.46) exhibited an ortho coupling (*J* = 9.2 Hz), consistent with their assignment as adjacent protons on a 1,2-disubstituted aromatic ring. In the HMBC spectrum, the position of the methoxy group was unequivocally assigned to C-3 (*δ_C_* 141.33) based on a clear long-range correlation from the methoxyl protons at *δ_H_* 3.92. Additional HMBC cross-peaks (Figure 4 and Figure S18) from H-2 (ring D) and H-4 (ring A) to the methoxy-substituted carbon at *δ_C_* 141.33 (C-3) provided definitive evidence for the placement of the methoxy group on ring D, while simultaneously confirming the proximity and fusion between rings A and D.

Critically, the position of the methoxy group was unequivocally assigned to C-3 (*δ_C_* 141.33) based on a long-range HMBC correlation from the methoxy protons (*δ_H_* 3.92). This regiochemistry was further supported by cross-peaks from H-2 (*δ_H_* 7.46) and H-4 (*δ_H_* 8.45) to C-3 (*δ_C_* 141.33), effectively excluding alternative positional isomers. The tetracyclic framework was confirmed by key HMBC correlations from H-1 to C-11b (*δ_C_* 126.38) and C-6b (*δ_C_* 147.15), and from H-5 to C-3a (*δ_C_* 114.30). These interactions establish the fusion between rings A, B, and D. These data, in strong agreement with literature for benzo[de]anthracen-7-one derivatives [13,14], confirm **HK2** as the new natural derivative product 6-hydroxy-3-methoxy-7H-benzo[*de*]anthracen-7-one.

### 2.3. Total Phenolic and Flavonoid Contents of T. teneriffae

Phenolic compounds are metabolites produced by plants, characterized by their distinctive aromatic structure and the presence of one or more -OH rings. Flavonoids, on the other hand, are a type of phenolic derivative pigment found in a wide range of higher plants. The total phenolic and flavonoid contents of the HAE from *T. teneriffae* are expressed in milligrams of gallic acid equivalent per gram of dried extract (mg GAE/g DE) and milligrams of quercetin equivalent per gram of dried extract (mg QE/g DE), respectively, and are summarized in Table 3. There were no previous studies dealing with the total phenolic and flavonoid contents determination in the hydroethanolic extract from *T. teneriffae* roots. However, compared with the aerial parts [10], the total phenolic content (101 ± 0.50 mg GAE/g) is similar to that in the roots (103.76 ± 0.67 mg GAE/g).

In contrast, the flavonoid content of the hydroethanolic root extract (112.97 ± 1.10 mg QE/g) is higher than that detected in the ethanolic extract of the aerial part (41 ± 1.60 mg QE/g) [10]. These results highlight the abundance of phenolic compounds, particularly flavonoids, in *T. teneriffae* roots, suggesting that further in-depth studies on this plant are warranted.

### 2.4. Antioxidant Activity

Antioxidants play a crucial role in preventing various diseases and slowing down the aging process [15]. They work by inhibiting or delaying the oxidation process by blocking the initiation or propagation of oxidizing chain reactions [16]. In this study, we aimed to evaluate the antioxidant potential of HAE, its fractions, 11-hydroxy-12H-benzo[*a*]xanthen-12-one, and 6-hydroxy-3-methoxy-7H-benzo[*de*]anthracen-7-one from *T. teneriffae* roots. For this purpose, three different assays were used: DPPH radical-scavenging, ferric reducing power (FRAP), and total antioxidant capacity. Vitamin C was used as a positive standard, a well-known antioxidant.

### 2.5. DPPH Free Radical-Scavenging Activity

The antioxidant potential of the *T. teneriffae* root HAE was evaluated using the DPPH radical scavenging assay, which measures the ability of compounds to donate hydrogen atoms and neutralize DPPH radicals, resulting in the formation of stable DPPH-H. The activity was evaluated by determining the IC_50_ value, which represents the concentration required to inhibit 50% of DPPH radicals. As shown in Table 3, the HAE, fraction 3, and 6-hydroxy-3-methoxy-7H-benzo[*de*]anthracen-7-one exhibited the strongest radical scavenging activity, with IC_50_ values of 0.068, 0.097, and 0.055 mg/mL, respectively, compared to the standard vitamin C (IC_50_ = 0.03 mg/mL). Fractions 1, 2, 4 and 11-hydroxy-12H-benzo[*a*]xanthen-12-one showed moderate activity (IC_50_ = 0.680, 0.580, 0.102 and 0.520 mg/mL). The high activity of the root extract can be attributed to its high content of phenolic and flavonoid compounds. Xanthones, which are oxygen-containing heterocyclic compounds [17] with various substituents (such as hydroxyl, methoxyl, prenyl, and glycosyl groups), are known for their diverse biological properties, including antioxidant, antidiabetic, anti-inflammatory, and antitumor activities [18,19,20]. Previous studies [21,22] have shown that the number and arrangement of free hydroxyl groups heavily influence the antioxidant activity of hydroxylated xanthones. One crucial factor is the distance between these groups within the molecule, with longer hydrogen bonds typically resulting in weaker interactions [21]. This, in turn, makes it easier for hydrogen atoms to transfer and scavenge DPPH radicals [21].

However, 11-hydroxy-12H-benzo[*a*]xanthen-12-one (**HK1**) deviates from this general behavior because its hydroxyl group at position 11 lies in close proximity to the adjacent carbonyl. As reported in the literature [21], this arrangement promotes the formation of a short, strong intramolecular hydrogen bond, which reduces the ability of the phenolic O–H bond to undergo hydrogen atom transfer. Such intramolecular stabilization not only decreases the availability of the hydroxyl hydrogen but also alters the electronic distribution within the xanthone core. This structural constraint provides a clear rationale for **HK1**’s low of reactivity, as its electronic environment is unfavorable to hydrogen-atom transfer under the assay conditions.

In contrast, 6-hydroxy-3-methoxy-7H-benzo[*de*]anthracen-7-one, which has angularly fused aromatic rings and two electron-donating groups (-OH and -OCH_3_), demonstrated potent radical scavenging activity, highlighting the importance of both molecular conformation and donor substituents in modulating antioxidant efficiency.

### 2.6. Determination of Total Antioxidant Capacity and Reducing Power of T. teneriffae

The antioxidant potential of the samples was evaluated using both total antioxidant capacity (TAC) and ferric reducing antioxidant power (FRAP) assays. TAC, measured by the phosphomolybdenum method, is based on the reduction of Mo(VI) to Mo(V), which forms a green complex at 695 nm. The HAE, fraction 3, and 6-hydroxy-3-methoxy-7H-benzo[*de*]anthracen-7-one showed the highest antioxidant capacities (230.604, 210.310, and 187.652 mg gallic acid equivalents GAE/g extract, respectively) (Table 3), whereas fractions 1, 2, 4 and 11-hydroxy-12H-benzo[*a*]xanthen-12-one exhibited moderate activity. This indicates that TAC reflects the overall synergistic effects of components rather than those of single molecules [23].

**Figure 5:**
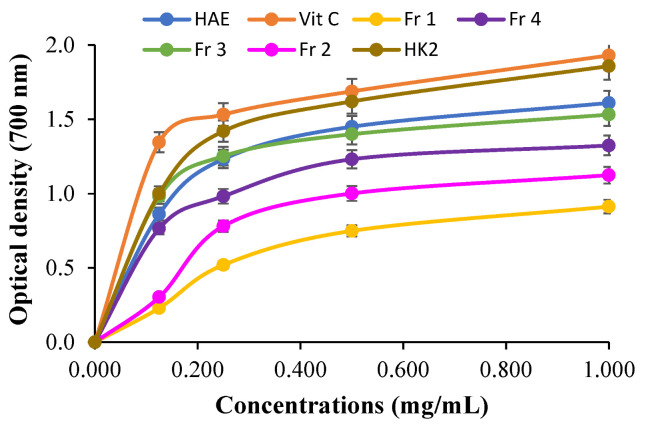
Reducing the power (OD700) of HAE extract, fractions, and isolated compounds of the root parts of *T. teneriffae*.

The FRAP assay evaluates electron-donating capacity by reducing Fe^3+^ to Fe^2+^. The reducing power increased with concentration [24] (Figure 5), with the HAE extract showing the strongest activity (*p* < 0.05), consistent with the TAC results. High phenolic and flavonoid contents, particularly 6-hydroxy-3-methoxy-7H-benzo[*de*]anthracen-7-one (**HK2**), likely contribute to this effect through electron transfer and hydrogen donation.

Overall, combining TAC and FRAP provides a comprehensive view of antioxidant potential: TAC captures cumulative, synergistic effects, while FRAP reflects direct electron-donating ability. The weak ability of 11-hydroxy-12H-benzo[*a*]xanthen-12-one to reduce Fe^3+^ to Fe^2+^ highlights the importance of molecular conformation and the stabilization of the molecule through resonance after electron donation in determining antioxidant efficiency. This finding confirms that 6-hydroxy-3-methoxy-7H-benzo[*de*]anthracen-7-one is the most potent antioxidant among the tested samples.

### 2.7. Molecular Docking Study

Excessive secretion of unbuffered gastric acid can cause damage to the stomach lining. This acid is produced by H^+^/K^+^-ATPase, an enzyme in the gastric parietal cells that pumps protons into the stomach in exchange for potassium ions. The proper function of this proton pump is important in the development of various types of ulcers. Because of this, H^+^/K^+^-ATPase is a key therapeutic target, and proton pump inhibitors have been developed to prevent and treat ulcer-related damage [25]. To understand how the extract works against ulcers, its two main compounds were tested in silico for their ability to bind and inhibit H^+^/K^+^-ATPase.

**Figure 6:**
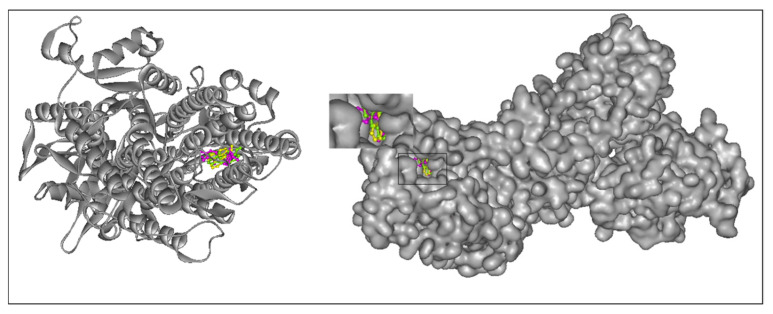
Best docking conformations of compounds **HK1** (green), **HK2** (yellow), and omeprazole (pink) within the active-site pocket of H^+^/K^+^-ATPase (PDB ID: 5YLU).

As shown in Figure 6, the tested compounds occupy the active-site cavity in a manner comparable to that of the standard reference molecule, omeprazole.

**Figure 7:**
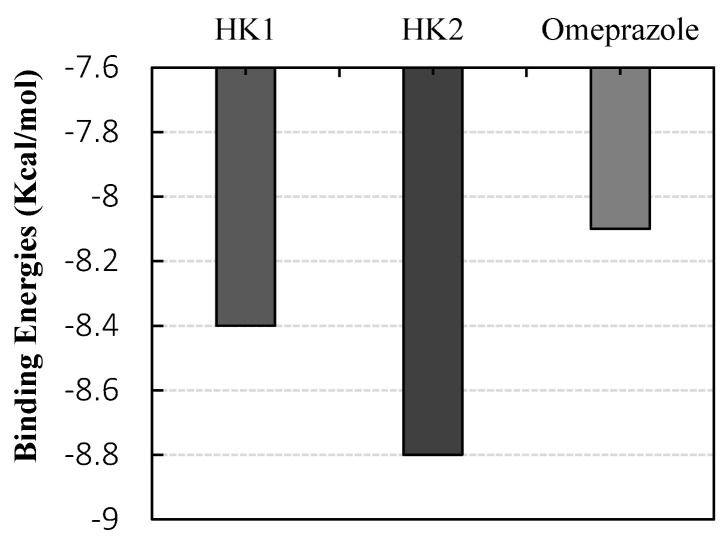
Binding energies of compounds **HK1**, **HK2**, and omeprazole within the active-site pocket of H^+^/K^+^-ATPase (PDB ID: 5YLU).

Results shown in Figure 6,suggest that **HK2** exhibited the strongest binding affinity toward the human H^+^/K^+^ ATPase, followed by **HK1**. In contrast, omeprazole showed the lowest affinity, with docking scores of −8.8, −8.4, and −8.1 kcal/mol, respectively (Figure 7).

**Figure 8:**
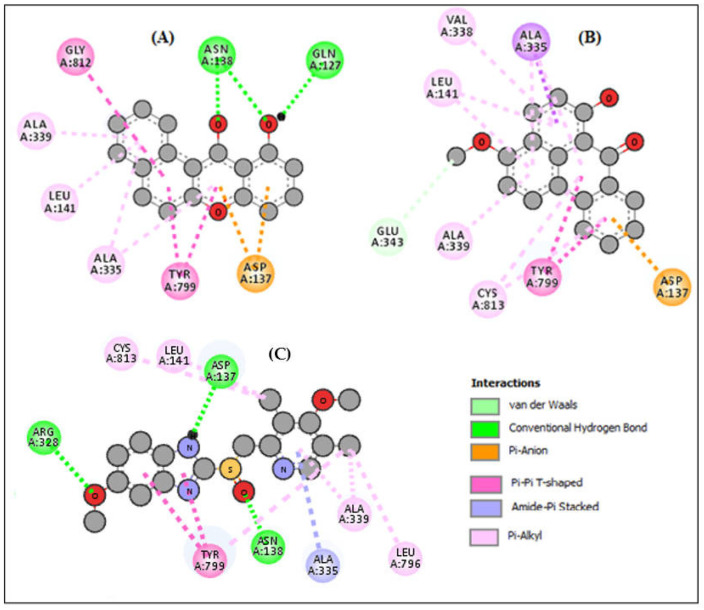
2D visualization of the molecular interaction of H^+^/K^+^-ATPase (PDB ID: 5YLU) with **HK1** (**A**), **HK2** (**B**), and omeprazole (**C**).

Figure 8 presents the 2D representations of the binding poses of the three tested compounds. Importantly, **HK2** (B) displayed the most favorable binding profile, characterized by an extensive hydrophobic bond interaction involving the key residues Ala335, Ala339, Leu141, and Val338, as well as π-alkyl interactions with Tyr799, which together form the hydrophobic core previously described as essential for the stabilization of potent proton-pump inhibitors [26].

This compound also formed a π-anion interaction with Asp137, similar to that observed in the omeprazole-H^+^/K^+^-ATPase complex, further reinforcing its strong binding affinity. **HK1** (A), although less strongly bound, exhibited a well-defined anchoring through two hydrogen bonds with Asn138 and Gln127, complemented by a π-anion interaction with Asp137 and seven hydrophobic interactions with Tyr 799, Gly 812, Ala 339, Leu 141, and Ala 141 and neighboring aliphatic residues. In contrast, the commercial treatment omeprazole (C) exhibited the weakest binding affinity, forming three hydrogen bonds with Arg328, Asn138, and Asp137, alongside seven hydrophobic interactions involving the same residues as observed in **HK1** and **HK2**. These computational insights correlate with the observed in vivo antisecretory effects and suggest that the gastroprotective activity of the isolated compounds may involve the modulation of the H^+^/K^+^-ATPase pump. Consequently, **HK2** emerges as a promising bioactive scaffold for further biochemical assays to confirm its potential as a natural modulator of gastric acid secretion.

### 2.8. Effect of GU and Compounds **HK1** and **HK2** on Gastric Inflammation and Oxidative Stress Markers

**Figure 9:**
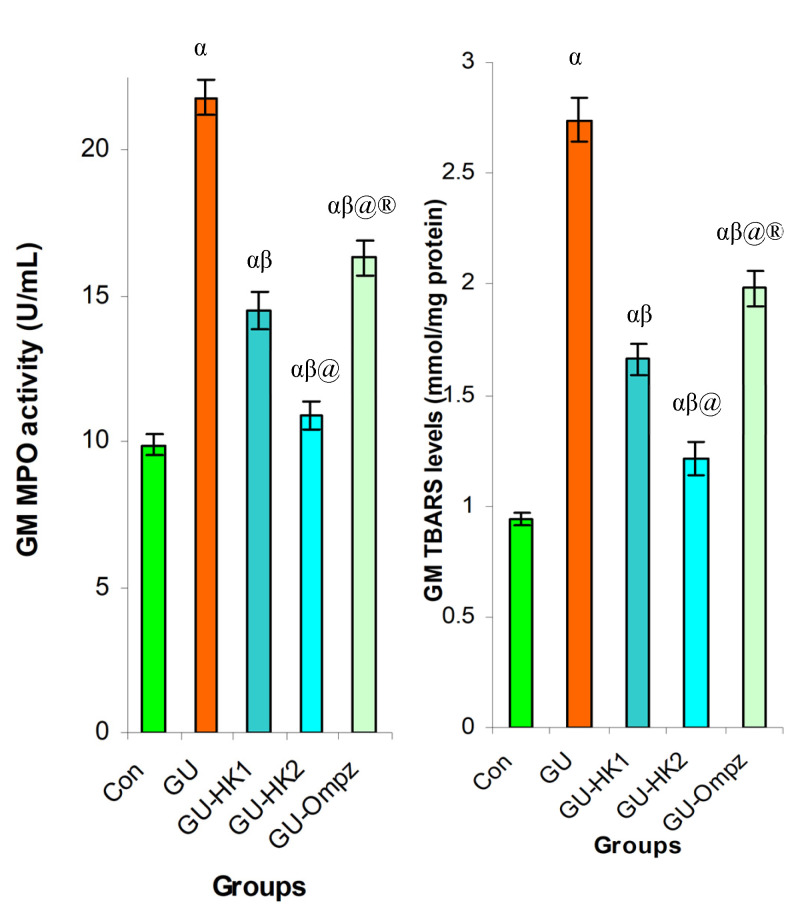
Effect of GU and the compounds **HK1** and **HK2** on MPO activity, H_2_O_2_ levels, and TBARS in the gastric mucosa. (α) treated groups versus the control group; (β) untreated GU group versus the control group; (@) GU-treated groups versus the untreated GU group; (^®^) GU-**HK1** versus GU-**HK2**.

The results illustrated in Figure 9, show that acute gastritis (GU) induced by oral administration of absolute ethanol leads to massive neutrophil infiltration in the gastric mucosa, as reflected by a marked increase in MPO activity (+120.87%) compared with healthy rats. This neutrophil infiltration is associated with significant inflammation and severe oxidative stress, as evidenced by elevated H_2_O_2_ levels (+131.2%) and increased TBARS (+191.4%) relative to the control group. Treatment with compounds **HK1**, **HK2**, and omeprazole (Ompz) significantly attenuated these alterations. Compared with the GU group, these interventions reduced neutrophil infiltration, as indicated by decreases in MPO activity (−33.5%, −50.0%, and −25.2% for GU-**HK1**, GU-**HK2**, and GU-Ompz, respectively), and led to notable reductions in H_2_O_2_ levels (−42.7%, −49.8%, and −23.7%) and TBARS (−39.4%, −55.8%, and −27.7%).

GU induces strong inflammation and oxidative stress, whereas **HK1** and particularly **HK2** significantly reduce these markers, demonstrating potent anti-inflammatory and antioxidant activities. All data are expressed as mean ± standard deviation (SD). Statistical comparisons were performed using one-way analysis of variance (ANOVA) followed by Tukey’s post hoc test for multiple comparisons. Differences were considered statistically significant at *p* ≤ 0.05. Specifically, comparisons were made as follows: (α) treated groups versus the control group; (β) untreated GU group versus the control group; (@) GU-treated groups versus the untreated GU group; (^®^) GU-**HK1** versus GU-**HK2**.

In fact, 11-hydroxy-12H-benzo[*a*]xanthen-12-one (**HK1**) and 6-hydroxy-3-methoxy-7H-benzo[*de*]anthracen-7-one (**HK2**) likely exert their gastroprotective effects through dual antioxidant and anti-inflammatory mechanisms, consistent with the known actions of dietary phenolics. Polyphenols are recognized for their ability to scavenge reactive oxygen species (ROS), reduce oxidative stress, and inhibit inflammatory cascades, due to their hydroxyl-rich structures that can donate electrons or a hydrogen atom [27,28,29].

In various ethanol-induced gastric ulcer models, polyphenolic extracts have been shown to suppress MPO activity, decrease lipid peroxidation (MDA/TBARS), and restore endogenous antioxidant defenses (SOD, CAT, GSH-Px), accompanied by improved mucosal integrity and reduced macroscopic lesions [30,31]. Additionally, polyphenols have been reported to modulate key cellular signaling pathways involved in mucosal defense, such as activating the Nrf2/HO-1 pathway and inhibiting pro-inflammatory MAPK/NF-κB pathways, leading to decreased expression of inflammatory mediators, reduced oxidative damage, and enhanced ulcer healing [32].

Furthermore, the antisecretory effects of polyphenols, including inhibition of gastric acid secretion, H^+^/K^+^-ATPase activity, and pepsin, provide an additional protective mechanism by reducing the aggressive action of gastric juice on the mucosa [33]. Several previous studies have demonstrated that xanthone-type polyphenols can exert significant gastroprotective effects in experimental ulcer models, supporting the relevance of the present findings. For example, pyranocycloartobiloxanthone A, a prenylated xanthone isolated from *Artocarpus obtusus*, markedly reduced ethanol-induced gastric lesions in rats, an effect associated with decreased myeloperoxidase activity, reduced lipid peroxidation, and restoration of endogenous antioxidants such as GSH and NP-SH [34]. Similarly, xanthones isolated from *Garcinia achachairu*, including prenylated and hydroxylated derivatives, were reported to attenuate gastric inflammation and oxidative stress by reducing MPO activity, malondialdehyde levels, and pro-inflammatory cytokines, while also inhibiting H^+^/K^+^-ATPase activity, suggesting a combined cytoprotective and antisecretory mechanism [35]. In addition, mangiferin, a glucosylated xanthone from *Mangifera indica*, demonstrated dose-dependent protection against both ethanol- and indomethacin-induced gastric ulcers, accompanied by reductions in gastric acid secretion and preservation of non-protein sulfhydryl levels [36]. More recently, α-mangostin was shown to alleviate ethanol-induced gastric injury through suppression of ROS generation, inhibition of MPO activity, and modulation of Nrf2/HO-1 and NF-κB signaling pathways, highlighting the importance of antioxidant and anti-inflammatory mechanisms in xanthone-mediated gastroprotection [37]. Although studies specifically addressing anthracenone derivatives remain limited, anthraquinone-rich polyphenolic complexes isolated from *Rumex tianschanicus*, containing compounds such as emodin and chrysophanol, have been reported to significantly reduce indomethacin-induced gastric lesions and inflammatory infiltration, supporting the contribution of polycycli anthracene-related phenolics to gastric mucosal defense [38]. Taken together, these reports indicate that while xanthone and anthracene-derived scaffolds are well known, their gastroprotective efficacy strongly depends on substitution patterns and molecular context, placing **HK1** and particularly **HK2** within a growing class of bioactive polyphenols with potent anti-inflammatory and antioxidant effects in gastric ulcer models.

### 2.9. Effect of **HK1** and **HK2** on Key Gastric Enzymes

**Figure 10:**
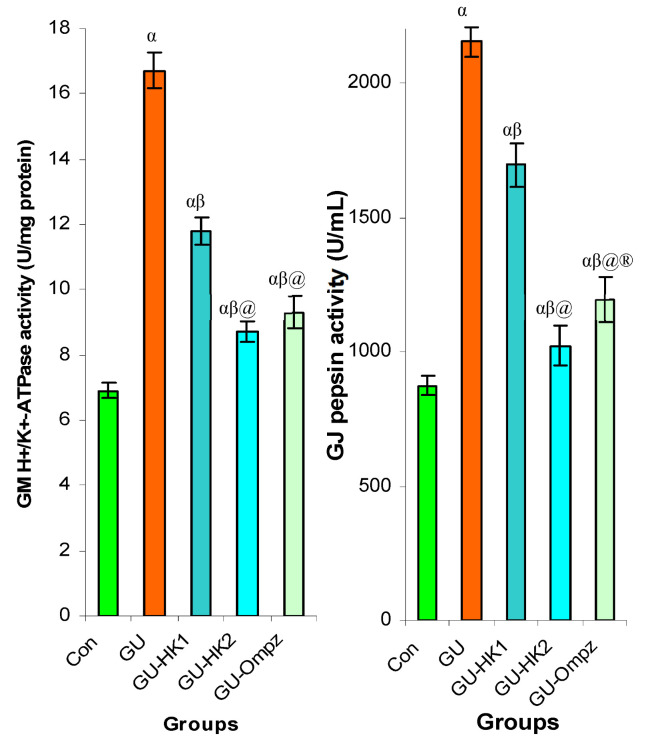
Effect of **HK1** and **HK2** on key gastric enzymes, including H^+^/K^+^-ATPase and pepsin, in the gastric mucosa. (α) treated groups versus the control group; (β) untreated GU group versus the control group; (@) GU-treated groups versus the untreated GU group; (^®^) GU-**HK1** versus GU-**HK2**.

Acute gastric ulceration induced by ethanol led to a marked increase in the activity of key gastric enzymes, including H^+^/K^+^-ATPase and pepsin, reflecting hypersecretion of gastric acid and proteolytic activity that contribute to mucosal damage. In the GU group (Figure 10), H^+^/K^+^-ATPase activity reached 16.7 U/mg protein and pepsin activity 2154 U/mL, significantly higher than in healthy controls. Treatment with **HK1** and **HK2** significantly reduced the activities of these enzymes, with **HK2** showing superior inhibitory effects (−47.9% for H^+^/K^+^-ATPase and −52.5% for pepsin) compared with **HK1** (−29.3% and −21.3%, respectively). These findings indicate that **HK1** and **HK2** exert their gastroprotective effects primarily by suppressing gastric acid secretion and pepsin hypersecretion.

Treatment with **HK1** and **HK2** significantly reduced the activities of these enzymes elevated by gastric ulceration, with **HK2** showing the strongest inhibitory effect, indicating their gastroprotective potential through suppression of acid and proteolytic hypersecretion. All data are expressed as mean ± standard deviation (SD). Statistical comparisons were performed using one-way analysis of variance (ANOVA) followed by Tukey’s post hoc test for multiple comparisons. Differences were considered statistically significant at *p* ≤ 0.05. Specifically, comparisons were made as follows: (α) treated groups versus the control group; (β) untreated GU group versus the control group; (@) GU-treated groups versus the untreated GU group; (^®^) GU-**HK1** versus GU-**HK2**.

The gastroprotective effects of **HK1** and **HK2** are further supported by their strong interactions with key gastric enzymes involved in ulcer formation, namely H^+^/K^+^-ATPase and pepsin. Molecular docking studies revealed that **HK1** and **HK2** bind efficiently to H^+^/K^+^-ATPase, with binding energies of −8.4 and −8.8 kcal/mol, respectively, compared to −8.1 kcal/mol for omeprazole, a standard proton pump inhibitor. These results indicate a high affinity of the polyphenolic compounds for this critical enzyme, suggesting potent inhibition of gastric acid secretion. In parallel, both **HK1** and **HK2** effectively modulated pepsin activity, thereby reducing proteolytic damage to the gastric mucosa. The combination of enzyme inhibition and the previously observed antioxidant and anti-inflammatory effects (reduced MPO, H_2_O_2_, and TBARS) underscores the multifactorial gastroprotective potential of **HK1** and **HK2**, with **HK2** demonstrating particularly strong efficacy.

### 2.10. Effect of **HK1**, **HK2**, and Omeprazole on Gastric Ulcer Indices

**Figure 11:**
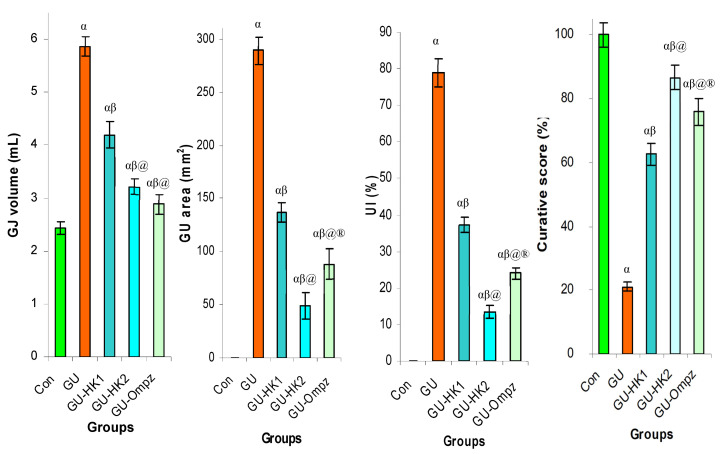
Effect of **HK1**, **HK2**, and omeprazole on gastric ulcer indices, including gastric juice (GJ) volume, ulcer area (GU area), ulcer index (UI), and curative score. (α) treated groups versus the control group; (β) untreated GU group versus the control group; (@) GU-treated groups versus the untreated GU group; (^®^) GU-**HK1** versus GU-**HK2**.

Acute gastric ulceration induced by ethanol significantly altered gastric parameters. As reported in Figure 11, In the GU group, gastric juice volume reached 5.87 mL, the total ulcer area was 289 mm^2^, the ulcer index (UI) was 79.0, and the curative score was 21.0%, reflecting severe mucosal damage. Treatment with **HK1**, **HK2**, and omeprazole markedly improved these indices. **HK1** reduced gastric juice volume, ulcer area, UI, and increased the curative score to 62.6%, while **HK2** showed superior protective effects, with ulcer area 49 mm^2^, UI 13.4, and curative score 86.6%. Omeprazole also significantly ameliorated these parameters, with an ulcer area of 88 mm^2^, UI of 24.0, and a curative score of 76.0%. These results highlight the gastroprotective efficacy of **HK1** and **HK2**, particularly **HK2**, in reducing gastric juice secretion and ulcer formation.

Gastric ulceration significantly increased these parameters, while treatment with **HK1** and **HK2** markedly improved them, with **HK2** showing the most pronounced gastroprotective effect. All data are expressed as mean ± standard deviation (SD). Statistical comparisons were performed using one-way analysis of variance (ANOVA) followed by Tukey’s post hoc test for multiple comparisons. Differences were considered statistically significant at *p* ≤ 0.05. Specifically, comparisons were made as follows: (α) treated groups versus the control group; (β) untreated GU group versus the control group; (@) GU-treated groups versus the untreated GU group; (^®^) GU-**HK1** versus GU-**HK2**.

Our findings suggest that **HK1** and **HK2** significantly improved ulcer parameters (gastric juice volume, ulcer area, ulcer index, and curative score) strongly suggest a potent gastroprotective action. These results resonate with earlier reports on polyphenolic compounds, in which ethanol-induced gastric injury was mitigated by reduced acid secretion, decreased oxidative stress, and lower neutrophil infiltration. For instance, extracts rich in phenolics were shown to suppress gastric lesions, lower lipid peroxidation (MDA/TBARS), and restore antioxidant defenses in models of ethanol-induced ulceration [39]. Furthermore, studies such as one on lycopene demonstrated attenuation of mucosal damage via reducing inflammatory mediators, such as MPO, PLA2, and ELA activities [40], and oxidative stress, together with protection of mucus and increased gastric pH effects similar to those observed with **HK1**/**HK2**. Importantly, our molecular docking data complement these experimental findings.

The binding energies for **HK1** (−8.4 kcal/mol) and **HK2** (−8.8 kcal/mol) with H^+^/K^+^-ATPase are more favorable than that of the commonly used proton-pump inhibitor Omeprazole (−8.1 kcal/mol), suggesting that **HK1** and **HK2** may directly inhibit the enzyme responsible for gastric acid secretion. This strong in silico interaction supports the observed reduction in gastric juice volume and ulcer severity. Similar mechanisms have been proposed in recent studies of plant polyphenols, which combined antioxidant, anti-inflammatory, and antisecretory actions to protect gastric mucosa.

### 2.11. Effect of **HK1** and **HK2** on Histological Evaluation of Gastric Damage

**Figure 12:**
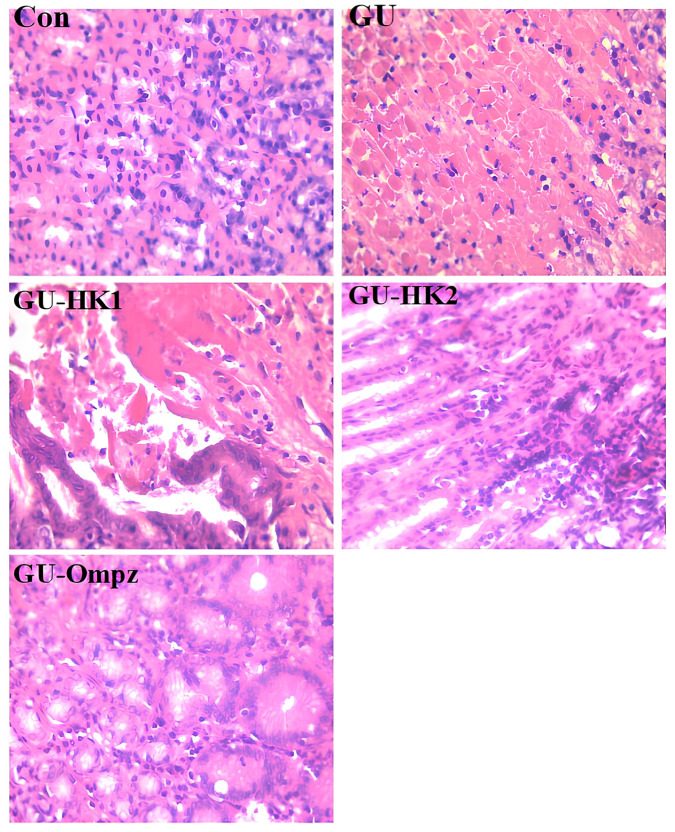
Photos from the pathology slides showing the effect of the administration of **HK1** or **HK2** to the gastric mucosa ulcer in rats.

In comparison to the gastric mucosa of normal rats, this study demonstrated that supplementing ethanol by the gastric gavage route caused a variety of lesions in the stomach of rats, including severe disruption of the gastric mucosa, flattening of the gastric mucosa, and necrotic lesions that deeply penetrated the mucosa and sub-mucosal layers (Figure 12). Furthermore, we demonstrated that omeprazole, **HK1,** or **HK2** prevented gastrointestinal mucosal ulcers, flattened gastric mucosa, and necrotic lesions in rats’ stomachs. Ingestion of **HK1** or **HK2** markedly protected ulcerated rats from the severe disruption of the gastric wall induced by ethanol, preventing haemorrhagic lesions and reducing the extent of ulceration (Figure 12). Supplementation with **HK1** or **HK2** also attenuated gastric inflammation, as evidenced by a strong inhibition of MPO activity and reduced H_2_O_2_ and TBARS levels. To the best of our knowledge, no previous studies have reported the anti-ulcer activity of the xanthenone **HK1** and anthracenone **HK2**.

Nevertheless, the strong gastroprotective effects observed in the present investigation are likely attributable to their potent anti-inflammatory and antioxidant properties, particularly their ability to inhibit neutrophil infiltration and recruitment, which are key contributors to ethanol-induced gastric injury. In addition, the strong interactions of **HK1** and **HK2** with key gastric enzymes—especially H^+^/K^+^-ATPase and pepsin—together with their high binding affinities, support their capacity to suppress gastric acidity and proteolytic activity, thereby enhancing mucosal protection and accelerating ulcer healing. These findings are consistent with previous investigations that have shown similar protective actions of polyphenolic compounds. For instance, Bahrini in 2025 [27] demonstrated that pre-treatment with astaxanthin prevented ethanol-induced micro- and macroscopic mucosal alterations. Abdella in 2025 [40] reported that raw olive leaf extract protected ethanol-treated rats from hemorrhagic mucosal lesions, decreased ulcer area, and reduced inflammatory mediators. Several studies have also highlighted the protective roles of bioactive phytochemicals in inflammatory conditions. Rezagholizadeh in 2017 [39] showed that oleuropein inhibits inflammatory enzymes, such as lipoxygenases, and suppresses pro-inflammatory cytokines (IL-6, TNF-α, IL-1β), while scavenging superoxide anions, neutralizing hypochlorous acid-derived radicals, and modulating MAPK signaling. Taken together, these findings corroborate our results and suggest that **HK1**, and particularly **HK2**, exerts comparable gastroprotective effects through a combination of anti-inflammatory, antioxidant, anti-apoptotic, and mucosa-preserving mechanisms.

There is severe disruption of the gastric mucosa, and necrotic lesions penetrating deeply into the mucosa and neutrophil infiltration in ulcer rats as compared to the gastric mucosa of normal rats (H&E stain, 400×).

## 3. Materials and Methods

### 3.1. Plant Material

Roots of *T. teneriffae* were harvested in April 2023 from Bou-Hedma National Park (Sidi Bouzid, Southern Tunisia; 33°30′ N, 9°38′ E). The botanical identification was formally conducted by Dr. Imed Mezghani (Faculty of Sciences of Sfax, Tunisia). A voucher specimen (LCSN-TT-2023) was deposited in the Herbarium of the Organic Chemistry 514 Laboratory (Natural Products Unit), Faculty of Sciences of Sfax, University of Sfax, Tunisia. The collected material was shade-dried at ambient temperature until a constant weight was reached (approx. 20 days). The dried roots were then pulverized into a fine, homogeneous powder and stored in a dark, dry environment at 22–25 °C until further analysis.

### 3.2. Extraction and Isolation of the Hydroalcoholic Extract of T. teneriffae

The dried powder of *T. teneriffae* root parts (100 g) was extracted by maceration in 600 mL of 70% ethanol (water solution (*v*/*v*)) at room temperature for 72 h. The obtained solutions were filtered, and each solvent was evaporated under reduced pressure in a rotary evaporator at 40 °C. Finally, the obtained extracts were kept in the dark at 4 °C until further analysis. The hydroalcoholic extract (HAE) (2.8 g), which showed the highest antioxidant and anti-ulcer activities, was subjected to a silica gel Column Chromatography (CC). Elution was performed using a polarity gradient starting with hexane–EtOAc (100:0 → 0:100), followed by a final Methanol (MeOH) wash. Based on Thin-Layer Chromatography (TLC) profiling, four fractions (Frs. 1–4) were collected. The four collected fractions (Frs. 1–4) were assessed for their antioxidant activity. Fraction 3 (150 mg), which exhibited the highest antioxidant activity, was further purified by Preparative TLC (Prep-TLC) using a hexane–dichloromethane (40:60) solvent system, yielding two pure compounds (**HK1** and **HK2**).

### 3.3. Structure Elucidation

The chemical structures of **HK1** and **HK2** were characterized using high-resolution mass spectrometry (HRESIMS) and infrared (IR) spectroscopy. Their structures were further elucidated using 1D and 2D NMR spectroscopy (^1^H, ^13^C NMR, HSQC, H-H COSY, and HMBC), providing detailed information on the molecular formula, functional groups, and proton and carbon environments of the compounds.

### 3.4. General Experimental Procedures

1D and 2D NMR spectra for **HK1** and **HK2** were recorded on a Bruker Avance III 400 MHz spectrometer (Bruker BioSpin GmbH, Rheinstetten, Germany) operating at 400 MHz for (^1^H) and 100 MHz for (^13^C). The instrument was equipped with a 5 mm PABBO BB probe. For **HK1** (in CDCl_3_), ^13^C NMR data were acquired using the zgpg30 pulse sequence with 5120 scans, a relaxation delay (D1) of 2.0 s, and a spectral width of 238.9 ppm. For **HK2** (in DMSO-d_6_), ^1^H NMR data were obtained via the zg30 pulse sequence with 16 scans, a relaxation delay of 1.0 s, and an extended spectral width of 20 ppm to ensure the detection of all exchangeable protons.

2D NMR experiments (COSY, HSQC, and HMBC) were performed using standard Bruker pulse programs (cosygp, hsqcedetgp, and hmbcgplpnd) with optimized acquisition parameters. All spectra were processed and analyzed using TopSpin 3.2 software. UV/VIS absorption spectra were recorded on a Helios Omega UV–visible spectrophotometer (Thermo Fisher Scientific, Waltham, MA, USA). IR spectra were determined on a PerkinElmer Spectrum 100 FT-IR spectrometer (PerkinElmer, Waltham, MA, USA) in a diamond disk. Silica gel (70–230 mesh, Sigma-Aldrich, St. Louis, MO, USA) was used for column chromatography (CC). High-resolution mass spectrometry (HR-MS) was recorded on a Waters Xevo G2-XS QToF spectrometer (Waters Corp., Milford, MA, USA) coupled with an HPLC Acquity H-Class (Waters Corp., Milford, MA, USA) for ESI. Absorbance was acquired in a spectrophotometer (Thermo Fisher Scientific, Spectronic 200E, Waltham, MA, USA). Organic solvents were distilled before use.

**11-hydroxy-12H-benzo[*a*]xanthen-12-one** (**HK1):** Yellow powder (30 mg); UV (CH_2_Cl_2_) *λ_max_* 238, 316, 383 nm; IR (cm^−1^) 1640 (conjugated C=O), 1611, 1588 (aromatic C=C), 1239 (C–O–C) and 3228 (O–H); ^1^H NMR (400 MHz, CDCl_3_): *δ_H_* 13.29 (s, 1H, 1-OH), 9.97 (d, *J* = 8.8 Hz, 1H, H-1), 8.18 (d, *J* = 9.2 Hz, 1H, H-5), 7.94 (d, *J* = 8 Hz, 1H, H-4), 7.82 (m, 1H, H-2), 7.64 (m, 1H, H-3), 7.62 (m, 1H, H-9), 7.56 (d, *J* = 9.2 Hz, 1H, H-6), 7.02 (m, 1H, H-8) and 6.88 (d, *J* = 8 Hz, 1H, H-10). ^13^C NMR (100 MHz, CDCl_3_): *δ_C_* 184.23 (C-12), 161.93 (C-11), 157.95 (C-6a), 155.09 (C-7a), 137.66 (C-5), 135.87 (C-9), 130.71 (C-4a), 130.16 (C-4b), 129.83 (C-2), 128.65 (C-4), 126.75 (C-1), 126.38 (C-3), 117.78 (C-6), 113.39 (C-12a), 110.86 (C-10), 110.37 (C-11a), 106.50 (C-8); HRESIMS *m*/*z* 263.0729 [M + H]^+^, (calcd for C_17_H_11_O_3_, 263.0708).

**6-hydroxy-3-methoxy-7H-benzo[*de*]anthracen-7-one (HK2):** Yellow powder (52 mg); UV (CH_2_Cl_2_) *λ_max_* 234, 260, 302, 325 nm; IR (cm^−1^) 1610 (conjugated C=O), 1600, 1565 (aromatic C=C), 2917 (OCH_3_), 1233 (C–O–C) and 3178 (O–H); ^1^H NMR (400 MHz, DMSO-d_6_): *δ_H_* 9.93 (s, 1H, 6-OH), 9.60 (d, *J* = 9.2 Hz, 1H, H-1), 8.45 (d, *J* = 9.2 Hz, 1H, H-4), 8.28 (d, *J* = 8.4, 1H, H-8), 7.87 (m, 1H, H-10), 7.73 (m, 1H, H-11), 7.70 (m, 1H, H-5), 7.53 (m 1H, H-9), 7.46 (d, *J* = 9.2 Hz, 1H, H-2) and 3.92 (s, 3H, 3-OCH_3_). ^13^C NMR (100 MHz, DMSO-d_6_): *δ_C_* 177.85 (C-7), 156.23 (C-6), 123.18 (C-7a), 147.15 (C-6b), 141.33 (C-3), 125.07 (C-9), 130.31 (C-4), 126.42 (C-8), 135.06 (C-10), 126.38 (C-11b), 154.63 (C-11a), 122.86 (C-1), 122.39 (C-2), 118.69 (C-5), 118.26 (C-11), 61.24 (3-OCH_3_). HRESIMS *m*/*z* 277.0869 [M + H]^+^, (calcd for C_18_H_13_O_3_, 277.0865).

### 3.5. Phytochemical Composition

#### 3.5.1. Total Phenolic Content

The Folin–Ciocalteu method [41] was used to determine the total phenolic contents (TPC). Briefly, 1 mL of Folin–Ciocalteu reagent was added to a solution containing 1 mL of each extract (1 mg/mL) after 5 min. Next, 1 mL of saturated solution (Na_2_CO_3_/H_2_O) was added. The final mixture was incubated at 25 °C for 90 min, and then, the absorbance was measured at 725 nm. Total phenolic content was expressed in milligrams of gallic acid (GAE) equivalent per gram of extract.

#### 3.5.2. Total Flavonoid Content

Total flavonoid content (TFC) was determined according to the aluminium chloride colorimetric method. The total flavonoid content was also determined as previously described by Dalleli in 2024 [41] 1 mL of sample extract and 0.3 mL sodium nitrite (5%) were mixed thoroughly in properly labeled test tubes for 5 min. Subsequently, 0.3 mL of aluminum chloride (10%) was mixed, followed by the addition of sodium hydroxide (2 mL), after 6 min to stop the reaction. Absorbance was immediately measured at 510 nm against a blank using a spectrophotometer (Shimadzu UV-2700i, Shimadzu Corp., Kyoto, Japan). Quercetin was used as a standard, and the average TFC concentration for triplicate analysis was expressed as milligrams of quercetin equivalent per hundred grams of sample (mg QE/g extract) on a dry-weight basis. Data were presented as mean ± SD.

### 3.6. Antioxidant Activity

#### 3.6.1. Assay of DPPH Radical-Scavenging Activity

The antiradical potential of the HAE and the isolated compounds was assessed using the 2,2-Diphenyl-1-picrylhydrazyl (DPPH) radical scavenging assay, following the methodology established by Keskes in 2017 [42]. Briefly, 2 mL of DPPH was mixed with different concentrations (0.063, 0.125, 0.250, 0.500, and 1.00 mg/mL) of each sample (HAE or isolated compounds). After 30 min of incubation in the dark at room temperature, the Optical Densities (OD) were measured at 517 nm, using vitamin C as a positive control. The percentages of inhibition (PI%) were calculated using the following formula:

(PI%) = [(Ablank − Asample)/Ablank] × 100

where A_blank_ is the absorbance of the control reaction (containing all reagents except the studied sample), and A_sample_ is the absorbance of the studied sample. The extract concentration providing 50% inhibition (IC_50_) was determined from the graph of inhibition percentage versus extract concentration.

#### 3.6.2. Total Antioxidant Capacity Assay (TAC)

The total antioxidant capacity of each sample was evaluated as previously described by Soltani in 2025 [43]. This assay was based on the reduction of Mo(VI) to Mo(V) by the extract, followed by the subsequent formation of a green phosphate/Mo(V) complex at acidic pH. A volume of 0.1 mL of each extract (1 mg/mL) was combined with 1 mL of the reagent solution, which consisted of 28 mM sodium phosphate, 0.6 M sulfuric acid, and 4 mM ammonium molybdate. The tubes were capped and incubated in a boiling water bath at 95 °C for 90 min. After incubation, the samples were cooled to room temperature, and the absorbance was measured at 695 nm against a blank using a spectrophotometer (Shimadzu UV-2700i, Shimadzu Corp., Kyoto, Japan). The blank containing 1 mL of reagent solution and the appropriate volume of the same solvent used for the extract was incubated under the same conditions. The ascorbic acid was used as a positive control, and the total antioxidant capacity of the extract and isolated compounds was expressed in terms of ascorbic acid equivalent (mg AAE/g extract).

#### 3.6.3. Ferric Ion Reducing Antioxidant Power (FRAP) Assay

The FRAP assay assesses an antioxidant’s ability to convert the ferric 2,4,6-tripyridyl-s-triazine complex [Fe(III)-(TPTZ)_2_]_2_ to the vividly blue ferrous complex [Fe(II)-(TPTZ)_2_]_2_ using a previously reported method [41]. In total, 1 mL of various concentrations of each sample (0.063, 0.125, 0.250, 0.500, and 1.00 mg/mL) was added to a solution containing 2.5 mL of 0.2 M phosphate buffer (pH 6.6) and 2.5 mL of potassium ferricyanide (1% *w*/*v*). The mixture was then incubated at 50 °C for 20 min. Subsequently, the sample was centrifuged at 3000 rpm for 10 min. A 2.5 mL aliquot of the upper layer was mixed with 2.5 mL of water and 0.5 mL of a 0.1% (*w*/*v*) ferric chloride solution. After incubation for 10 min at room temperature, the absorbance of the reaction mixture was then read spectrophotometrically at 700 nm. A standard curve was prepared using vitamin C at various concentrations.

### 3.7. Experimental Study

The experimental protocol was approved by the National Ethics Committee (Approval Code: TNAPO-06-B-074; Approval Date: 23 December 2024). All animal procedures were conducted in accordance with international guidelines for the care and use of laboratory animals. Male Wistar rats, 80 days old and weighing 174 ± 8 g, were used to assess the anti-ulcer effects of **HK1** and **HK2**. Gastric ulcers were induced by orally administering 5 mL/kg of absolute ethanol via gastric gavage one hour after treatment with either **HK1**, **HK2**, or omeprazole. The animals were divided into five groups: a healthy control group (C), an untreated gastric ulcer group (GU), a GU group treated with **HK1** at 200 mg/kg body weight [27] (GU-**HK1**), a GU group treated with **HK2** at 200 mg/kg (GU-**HK2**), and a GU group treated with omeprazole at 20 mg/kg (GU-Ompz) [27]. After 90 min, the rats were sacrificed, and their stomachs were excised, opened, and thoroughly cleansed. The total ulcer area was measured using an inverted microscope with a digital camera and ImageJ software (version 1.53t, National Institutes of Health, Bethesda, MD, USA). Gastric mucosa content was measured, and the ulcer index (UI) was calculated with the formula: UI = (Ulcer area/Total mucosal surface area) × 100 [28]. The curative ratio was also determined: curative score = (UI control − UI treated)/UI control. Gastric juice was collected by centrifuging the gastric mucus, and its volume was measured using graduated tubes [30].

### 3.8. Analytical Methods

Animals were euthanized, and the esophagogastric junction was ligated before the stomachs were excised. Gastric mucus was drained and centrifuged at 3500× *g* for 5 min at 4 °C to remove insoluble components. The gastric mucosa was weighed in milligrams as an indicator of treatment-induced changes, and its content was determined according to the method of Ofusori in 2020 [31]. The volume of gastric juice was measured using graduated tubes. Pepsin activity was determined by incubating gastric juice with a casein buffer solution, 1 M trichloroacetic acid, 2% ninhydrin, and 1 mL sodium acetate buffer (2 M, pH 5.0), followed by absorbance measurement at 568 nm [32]. Lipid peroxidation (TBARS) was measured using the Buege and Aust method [15], mixing gastric juice with 0.5% thiobarbituric acid in 0.25 N HCl, heating at 95 °C for 30 min, and measuring absorbance at 532 nm. Protein concentration was assessed using the Biuret method. Myeloperoxidase (MPO) activity was measured spectrophotometrically at 460 nm using tetramethylbenzidine and H_2_O_2_ [44], with results expressed as μmol/mg protein/min. H_2_O_2_ levels in gastric juice were measured at 505 nm following the protocol of Dingeon in [45]. For H^+^/K^+^-ATPase activity, gastric mucosa was scraped, homogenized in Tris-HCl buffer (50 mM, pH 7.4) containing sucrose and EDTA, and centrifuged sequentially to isolate membrane fractions. The enzyme reaction was carried out with ATP, MgCl_2_, and KCl at 37 °C for 30 min, terminated with ammonium molybdate and perchloric acid, and inorganic phosphate release was measured at 400 nm. Pepsin activity was also assessed at 660 nm [46].

### 3.9. Statistical Analysis

The data were presented as mean ± standard deviation (SD) for at least three independent experiments (n = 3). Statistical analysis was performed using one-way analysis of variance (ANOVA) followed by Tukey’s post hoc test for multiple comparisons. Results were considered statistically significant at *p* < 0.05. Detailed significance levels for specific group comparisons (α, β, @, ^®^) are indicated in the respective figure legends.

### 3.10. Molecular Docking Studies

The molecular docking study of **HK1** and **HK2** from *T. teneriffae* was performed using AutoDock Vina (version 1.1.2), with post-docking visualization carried out using Discovery Studio Visualizer R2 and ViewerLite (version 5.0). The three-dimensional structure of human H^+^/K^+^-ATPase (PDB ID: 5YLU) was retrieved from the Protein Data Bank and prepared by removing water molecules and co-crystallized ligands [47], followed by the addition of polar hydrogen atoms and assignment of Kollman charges. The chemical structures of **HK1**, **HK2**, and the reference inhibitor omeprazole were drawn using ChemDraw (version 12.0). Docking simulations were conducted using a grid box of 40 × 40 × 40 Å centered on the binding site of the co-crystallized inhibitor vonoprazan (x = 136.43, y = 2.10, z = 12.06 Å). The protein was treated as rigid, while ligands were allowed full conformational flexibility. For each ligand, ten binding poses were generated with an exhaustiveness value of 8, and the optimal pose was selected based on the lowest predicted binding free energy and a favorable orientation within the active site.

## 4. Conclusions

The new natural derivative xantheneone and antharacenone compounds **HK1** and **HK2** isolated from HAE from *T. teneriffae* roots exhibit strong gastroprotective effects against ethanol-induced gastric ulceration. They significantly reduced ulcer area, gastric juice volume, and ulcer index while increasing the curative score, with **HK2** showing superior efficacy. These protective effects are mediated through inhibition of key gastric enzymes, H^+^/K^+^-ATPase and pepsin, as supported by strong molecular docking interactions. Additionally, **HK1** and **HK2** attenuated oxidative stress and neutrophil infiltration, lowering MPO, H_2_O_2_, and TBARS levels. The combined antisecretory, anti-proteolytic, antioxidant, and anti-inflammatory actions contribute to enhanced mucosal healing. **HK2**, in particular, demonstrated a multi-target mechanism comparable to or superior to omeprazole. Collectively, these findings demonstrate that **HK1** and **HK2** possess significant gastroprotective properties in this experimental model, likely through a multi-target mechanism involving antioxidant and anti-inflammatory pathways. While their interaction with H+/K^+^-ATPase and pepsin is supported by molecular docking, further experimental studies are needed to characterize their specific enzymatic inhibition and pharmacological profile.

## Figures and Tables

**Table 1:** ^1^H (400 MHz) and ^13^C (100 MHz) NMR assignments for **HK1** in CDCl_3_, including 2D correlations (COSY, HMBC) and comparison with literature data for 11-amino-12H-benzo[a]xanthen-12-one [12].

Position	HK1 *δ_C_* ppm	HSQC *δ_H_* ppm (m, *J* Hz)	H-H Cosy	HMBC	δ_C_ ppm (Lit. [12] *)
1	126.75	9.97 d (8.8)	H-2	C-3, C-4b, C-12a	127.4
2	129.83	7.82 m	-	C-4	130.1
3	126.38	7.64 m	H-4	C-2, C-1	126.9
4	128.65	7.94 d (8)	H-3	C-4a, C-4b	130.0
4a	130.71	-	-	-	132.0
4b	130.16	-	-	-	131.3
5	137.66	8.18 d (9.2)	H-6	C-6a, C-4a, C-4	137.1
6	117.78	7.56 d (9.2)	-	C-6a, C-12a	118.9
6a	157.95	-	-	-	158.9
7(CO)	-	-	-	-	-
7a	155.09	-	-	-	157.3
**8**	**106.50**	**7.02 d (8.4)**	**H-9**	**C-7a, C-11a**	**91.2**
**9**	**135.87**	**7.62 m**	-	**C-7a, C-11**	**164.6**
**10**	**110.86**	**6.88 d (8)**	-	**C-8, C-11, C-11a**	**97.0**
**11**	**161.93**	-	-	-	**154.7**
**11a**	**110.37**	-	-	-	**103.9**
12	184.23	-	-	-	181.2
12a	113.39	-	-	-	114.8
11-OH	-	13.29 (s)	-	-	-

* 11-Amino-9-hydroxy-12H-benzo[a]xanthen-12-one.

**Table 2:** ^1^H (400 MHz) and ^13^C (100 MHz) NMR assignments for **HK2** in DMSO-d_6_, including 2D correlations (COSY, HMBC).

Position	HK2 *δ_C_* ppm	HSQC *δ_H_* ppm (m, *J* Hz)	H-H Cosy	HMBC
1	122.86	9.60 d (9.2)	H-2	C-6b, C-11b
2	122.39	7.46 d (9.2)	H-1	C-3
3	141.33	-	-	-
3a	114.30	-	-	-
4	130.31	8.45 d (9.2)	H-5	C-6, C-3, C6a
5	118.69	7.70 m	H-4	C-3a, C-6
6	156.23	-	-	-
6a	124.82	-	-	-
6b	147.15	-	-	-
7	177.85	-	-	-
7a	123.18	-	-	-
8	126.42	8.28 d (8.4)	H-9	C-10, C-7, C-11a
9	125.07	7.53 m	H-10	C-11, C-7a
10	135.06	7.87 m	H-9	C-8, C-11a
11	118.26	7.73 m	-	C-7a, C-11a C-11b
11a	154.63	-	-	-
11b	126.38	-	-	-
3–OCH_3_	61.24	3.92 (s)	-	C-3
6–OH	-	9.93 (s)	-	-

**Table 3:** DPPH radical-scavenging activity and total antioxidant capacity (TAC) of HAE obtained fractions, and isolated compounds from *T. teneriffae* roots.

	DPPH IC_50_ (mg/mL)	TAC (mg GAE/g Extract)
HAE	0.068 ± 0.010	230.603 ± 0.610
Fr 1	0.680 ± 0.030	85.113 ± 0.130
Fr 2	0.580 ± 0.030	122.014 ± 0.338
Fr 3	0.097± 0.010	210.310 ± 0.588
Fr 4	0.102 ± 0.020	156.369 ± 0.444
**HK1**	0.520 ± 0.030	92.120 ± 0.140
**HK2**	0.055 ± 0.010	187.652 ± 0.534
Vitamin C	0.030 ± 0.002	249.520 ± 0.620

Values are presented as mean ± SD (*n* = 3). The differences were analyzed using an ANOVA test with multiple comparisons with *p* < 0.05.

## Data Availability

Data are contained within the article and Supplementary Materials.

## Supplementary Materials

The following supporting information can be downloaded at: https://www.mdpi.com/article/10.3390/molecules31111850/s1, Figure S1: HRESIMS Data (Positive mode) of compound 1; Figure S2: UV-Vis spectrum in CH_2_Cl_2_ of compound 1; Figure S3: FT-IR Spectrum of compound 1; Figure S4: ^1^H NMR spectrum (400 MHz, CDCl_3_) of compound 1; Figure S5: ^13^C NMR (100 MHz, CDCl_3_) of compound 1; Figure S6: DEPT spectrum of compound 1; Figure S7: HSQC spectrum of compound 1; Figure S8: HMBC spectrum of compound 1; Figure S9: H-H COSY spectrum of compound 1; Figure S10: HRESIMS Data (Positive mode) of compound 2; Figure S11: UV-Vis spectrum in CH_2_Cl_2_ of compound 2; Figure S12: FT-IR Spectrum of compound 2; Figure S13: ^13^C NMR (100 MHz, CDCl_3_) of compound 2; Figure S14: DEPT spectrum of compound 2; Figure S15: HSQC spectrum of compound 2; Figure S16: ^1^H NMR spectrum (400 MHz, CDCl_3_) of compound 2; Figure S17: H-H COSY spectrum of compound 2; Figure S18: HMBC spectrum of compound 2.

## Abbreviations

The following abbreviations are used in this manuscript:

CC	Column Chromatography
EtOA	Ethyl acetate
HAE	Hydroalcoholic Extract
H^+^/K^+^-ATPase	Hydrogen potassium ATPase (Proton pump)
H_2_O_2_	Hydrogen peroxide
MPO	Myeloperoxidase
ROS	Reactive Oxygen Species
TBARS	Thiobarbituric acid reactive substances
