# Supplementary material for: Investigation of Antioxidant, In Silico and In Vivo Antiulcer Activities of New Natural Xanthenone and Antracenone Isolated from Tricholaena teneriffae L. Roots
Source: Molecules. 2026 May 28;31(11):1850. doi: 10.3390/molecules31111850 (PMC13257519; doi:10.3390/molecules31111850)
Supplement: Supplementary file 1 [file molecules-31-01850-s001.zip › molecules-4109369-supplementary.pdf]

Investigation of Antioxidant, in Silico and in Vivo Antiulcer activities of New Natural Xanthenone and Antracenone Isolated from *Tricholaena teneriffae* L. Roots

Henda Keskes [a], Siwar Soltani [a], Khaled Hamden [b], Anthony Abou Dib [c], Jean-Hugues Renault [c], Musafau Sanni[d], Abdel Halim Harrath[d], Noureddine Allouche [a], * and Hichem Ben Salah [a]

[a] Laboratory of Organic Chemistry LR17ES08, Natural Substances Team, Faculty of Sciences of Sfax, University of Sfax, P.O. Box 1171, Sfax 3000, Tunisia
[b] Laboratory of Bioresources, Integrative Biology and Exploiting, Higher Institute of Biotechnology of Monastir, University of Monastir, Tunisia
[c] Université de Reims Champagne Ardenne, CNRS, ICMR UMR 7312, Reims, France
[d] Department of Zoology, College of Science, King Saud University, Riyadh, Saudi Arabia; hharrath@ksu.edu.sa
* Correspondence: noureddineallouche@yahoo.fr; noureddine.allouche@fss.usf.tn
Tel: (+216) 55 644 921

Supplementary Materials: Spectroscopic Characterization and HRESIMS Data for Compounds HK1 and HK2

Compound 1 (HK1): 11-hydroxy-12H-benzo[a]xanthen-12-one

Figure S1: HRESIMS Data (Positive mode) of compound 1.

Figure S2: UV-Vis spectrum in CH_2Cl_2 of compound 1.

Figure S3: FT-IR Spectrum of compound 1.

Figure S4: ^{1}H NMR spectrum (400 MHz, CDCl3) of compound 1.

Figure S5: ^{13}C NMR (100 MHz, CDCl3) of compound 1.

Figure S6: DEPT spectrum of compound 1.

Figure S7: HSQC spectrum of compound 1.

Figure S8: HMBC spectrum of compound 1.

Figure S9: H-H COSY spectrum of compound 1.

Compound 2 (HK2): 6-hydroxy-3-methoxy-7H-benzo[de]anthracen-7-one.

Figure S10: HRESIMS Data (Positive mode) of compound 2.

Figure S11: UV-Vis spectrum in CH_2Cl_2 of compound 2.

Figure S12: FT-IR Spectrum of compound 2.

Figure S13: ^{13}C NMR (100 MHz, CDCl3) of compound 2

Figure S14: DEPT spectrum of compound 2.

Figure S15: HSQC spectrum of compound 2.

Figure S16: ^{1}H NMR spectrum (400 MHz, CDCl3) of compound 2.

Figure S17: H-H COSY spectrum of compound 2.

Figure S18: HMBC spectrum of compound 2.

Figure S1

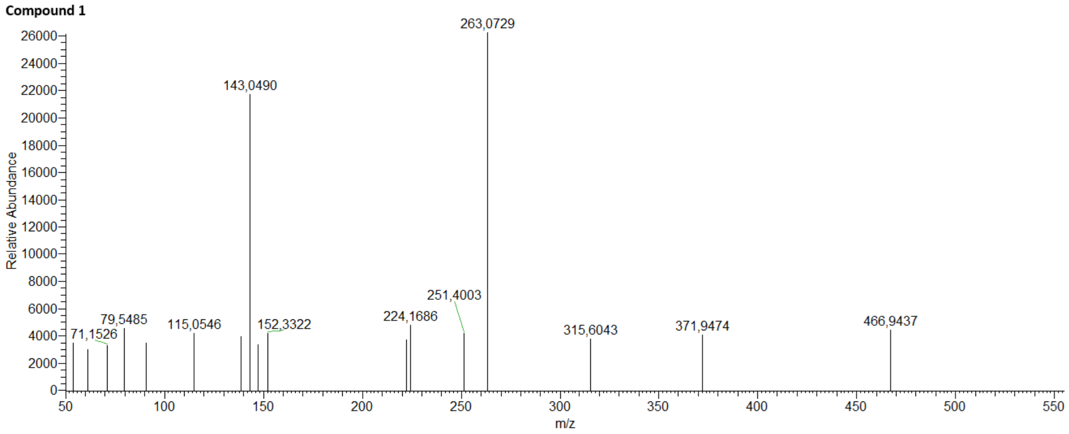

Figure S2

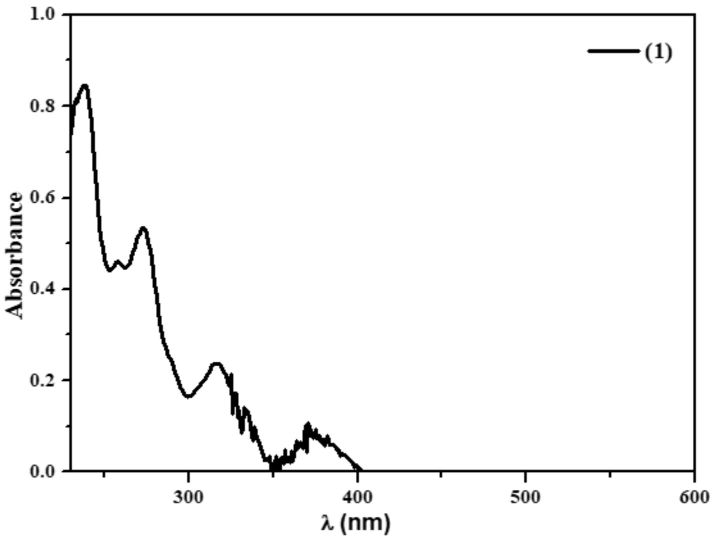

Figure S3

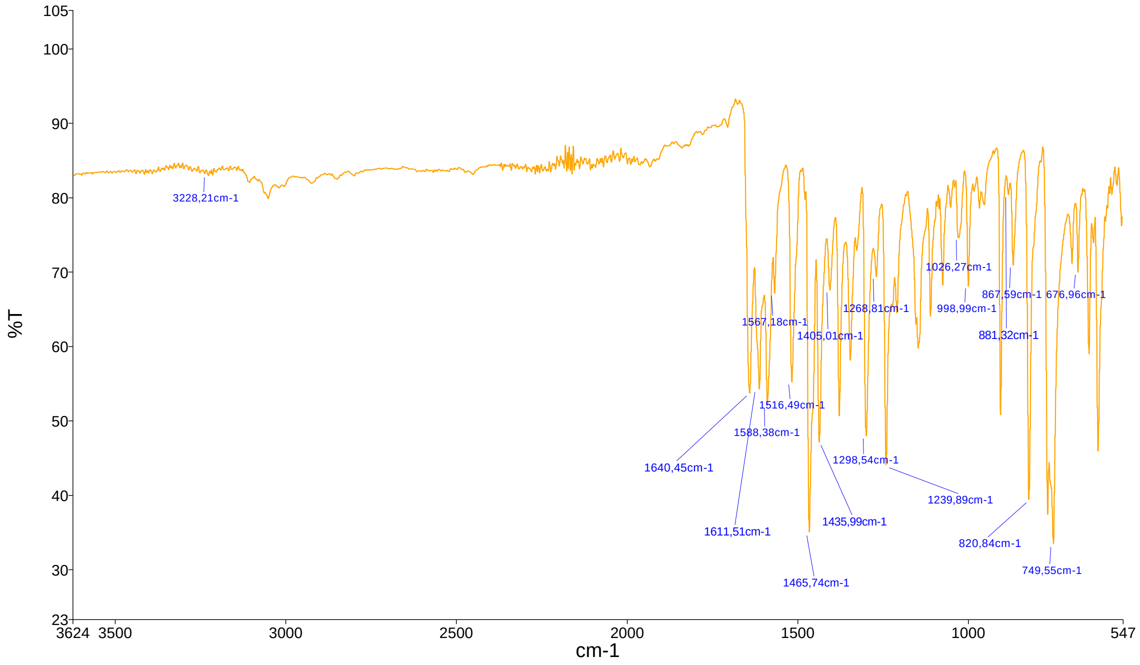

%T
cm-1
3228,21cm-1
1640,45cm-1
1611,51cm-1
1588,38cm-1
1567,18cm-1
1516,49cm-1
1465,74cm-1
1435,99cm-1
1405,01cm-1
1298,54cm-1
1268,81cm-1
1239,89cm-1
1026,27cm-1
998,99cm-1
881,32cm-1
867,59cm-1
820,84cm-1
749,55cm-1
676,96cm-1

Figure S4

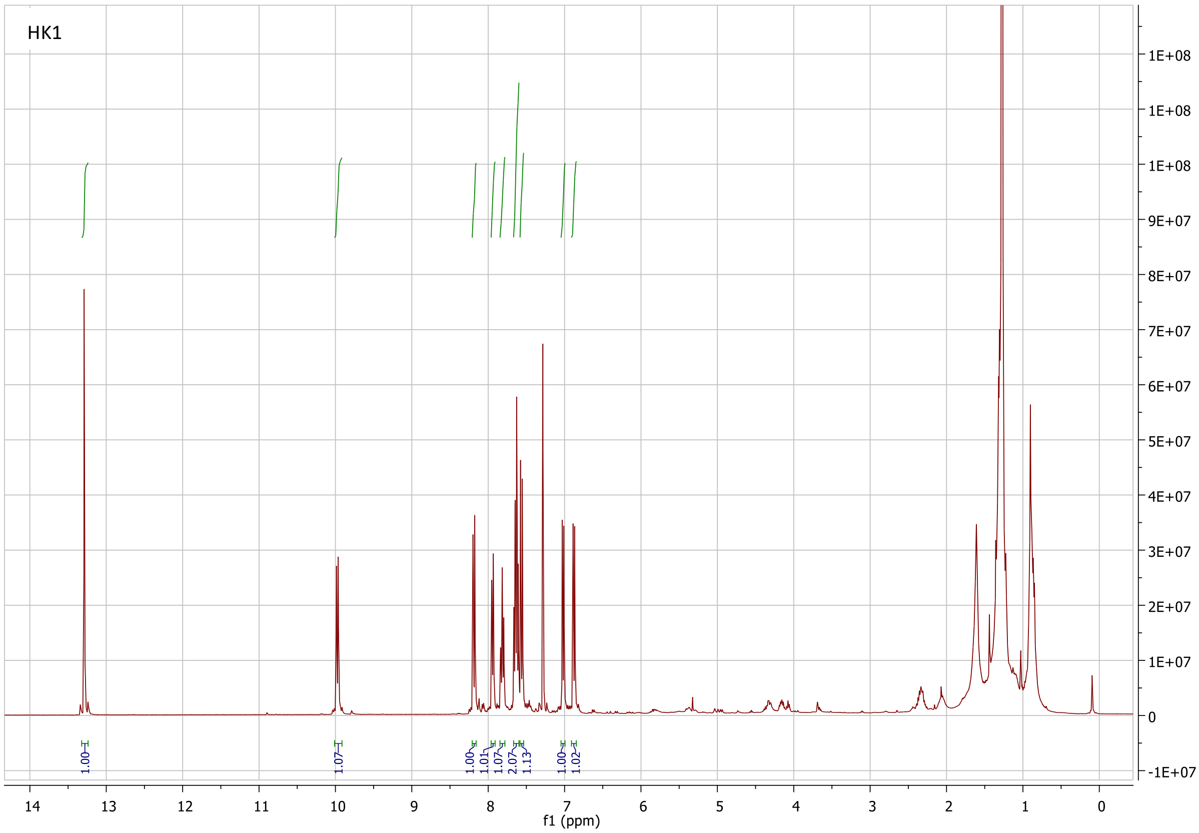
HK1
1.00
1.07
1.00
1.01
1.07
2.07
1.13
1.00
1.02
f1 (ppm)

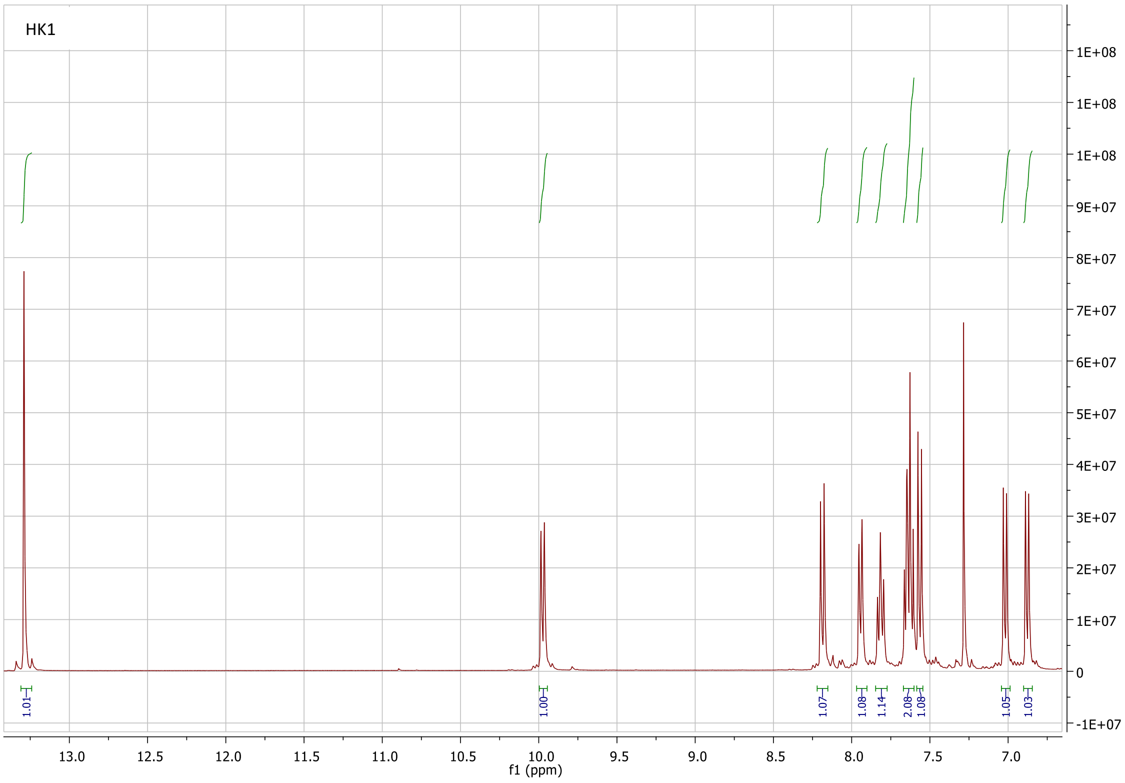
HK1
1.01
1.00
1.07
1.08
1.14
2.08
1.08
1.05
1.03
f1 (ppm)

Figure S5

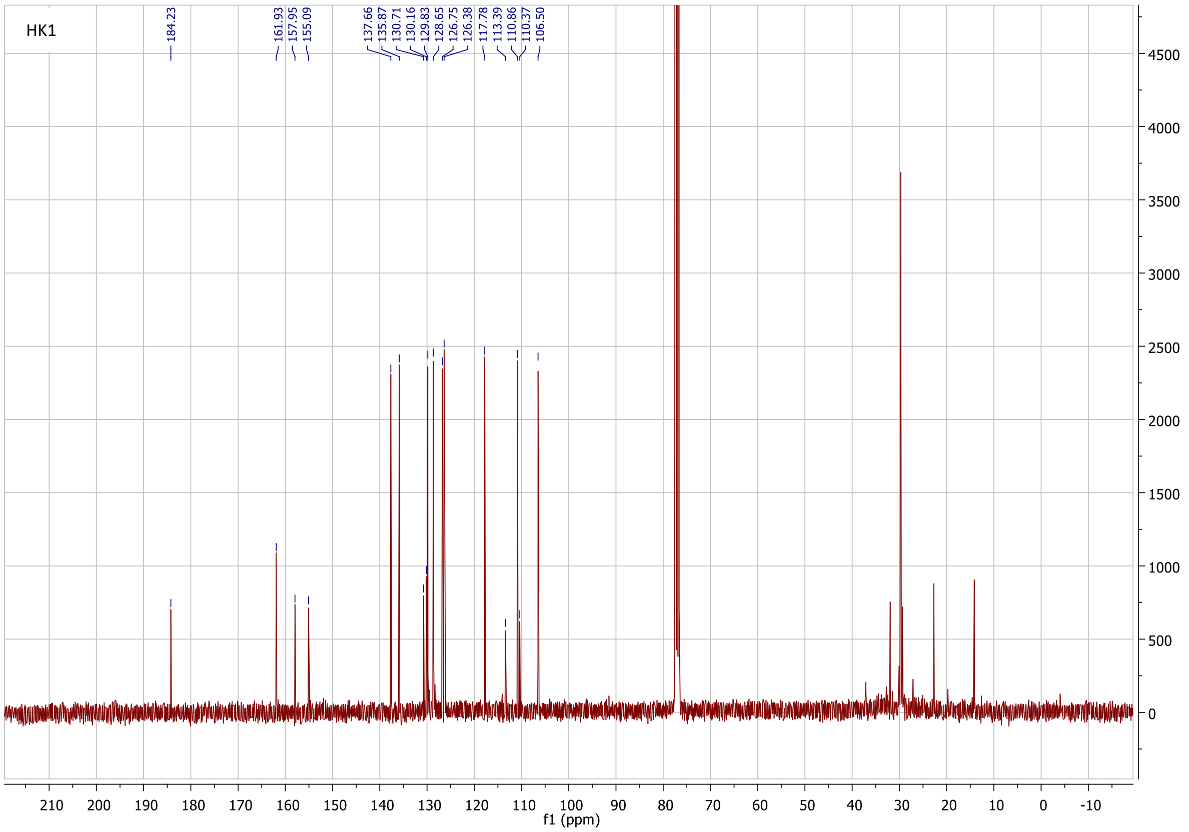
HK1
184.23
161.93
157.95
155.09
137.66
135.87
130.71
130.16
129.83
128.65
126.75
126.38
117.78
113.39
110.86
110.37
106.50
f1 (ppm)

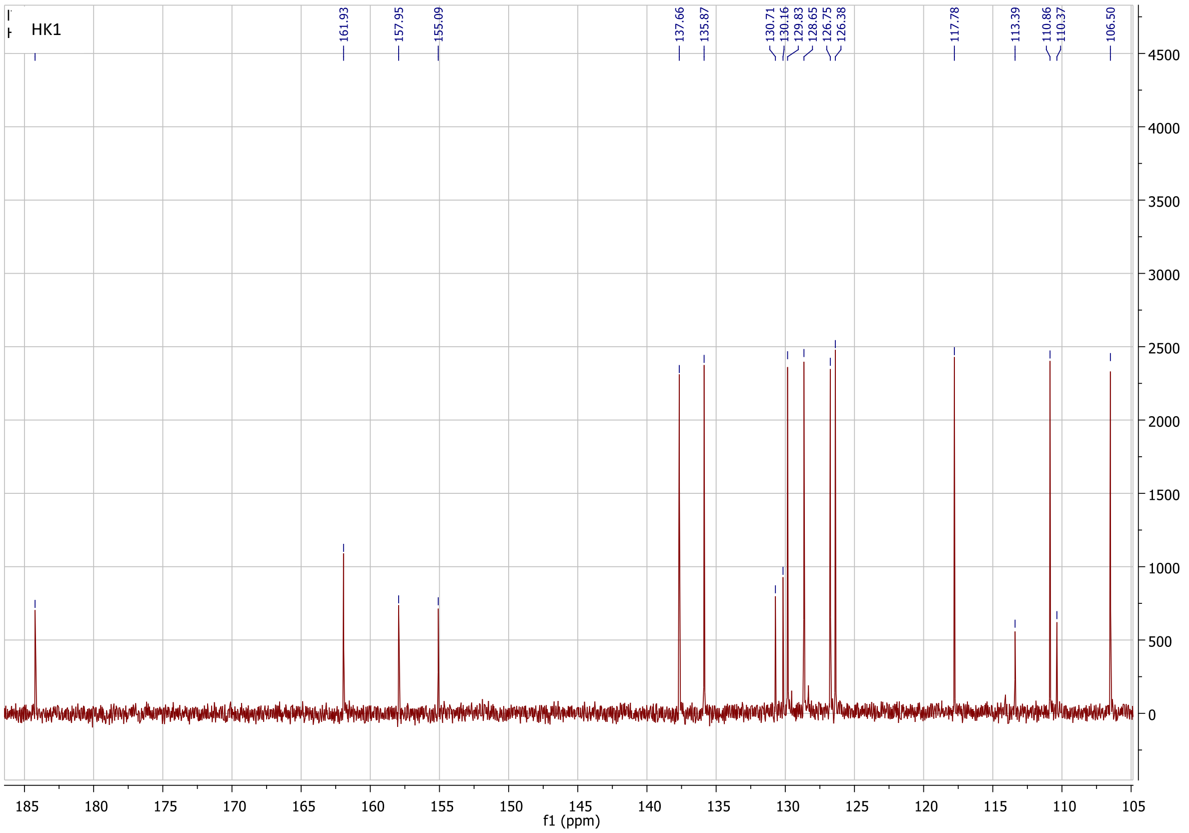
HK1
161.93
157.95
155.09
137.66
135.87
130.71
130.16
129.83
128.65
126.75
126.38
117.78
113.39
110.86
110.37
106.50
f1 (ppm)

Figure S6

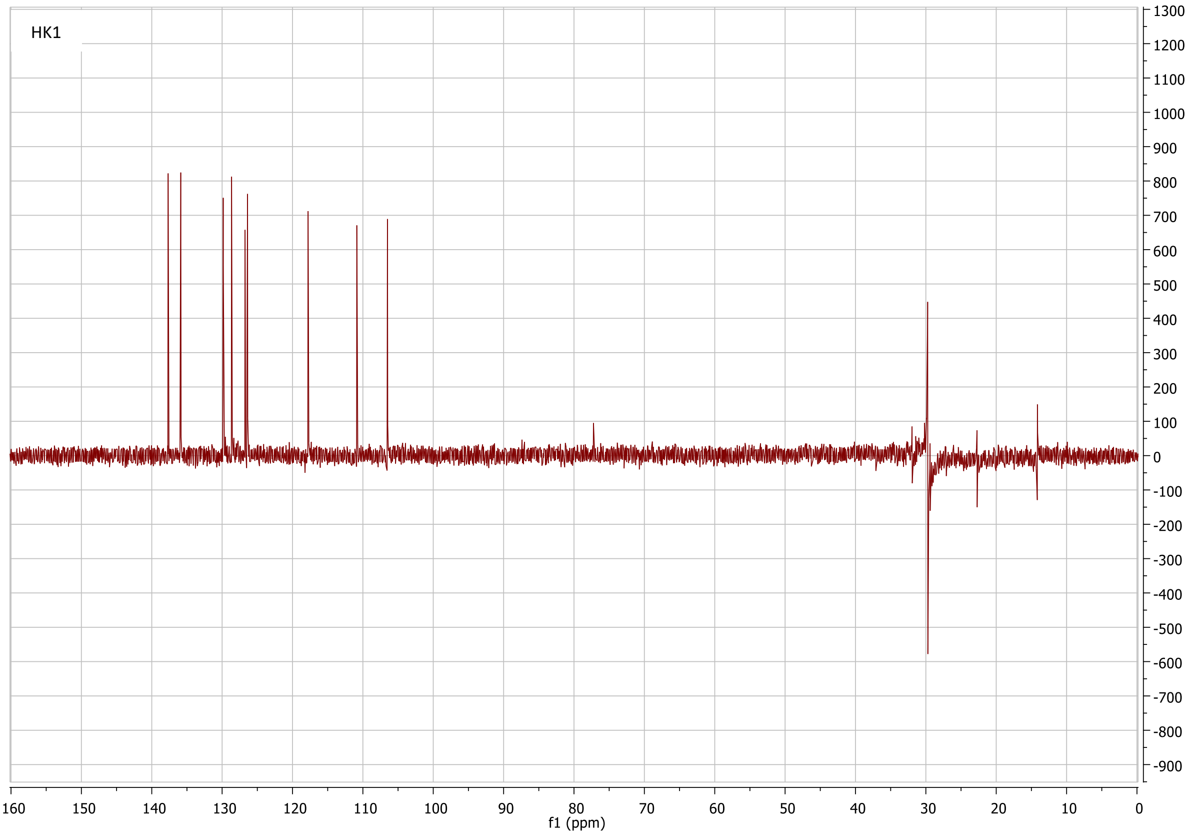

Figure S7

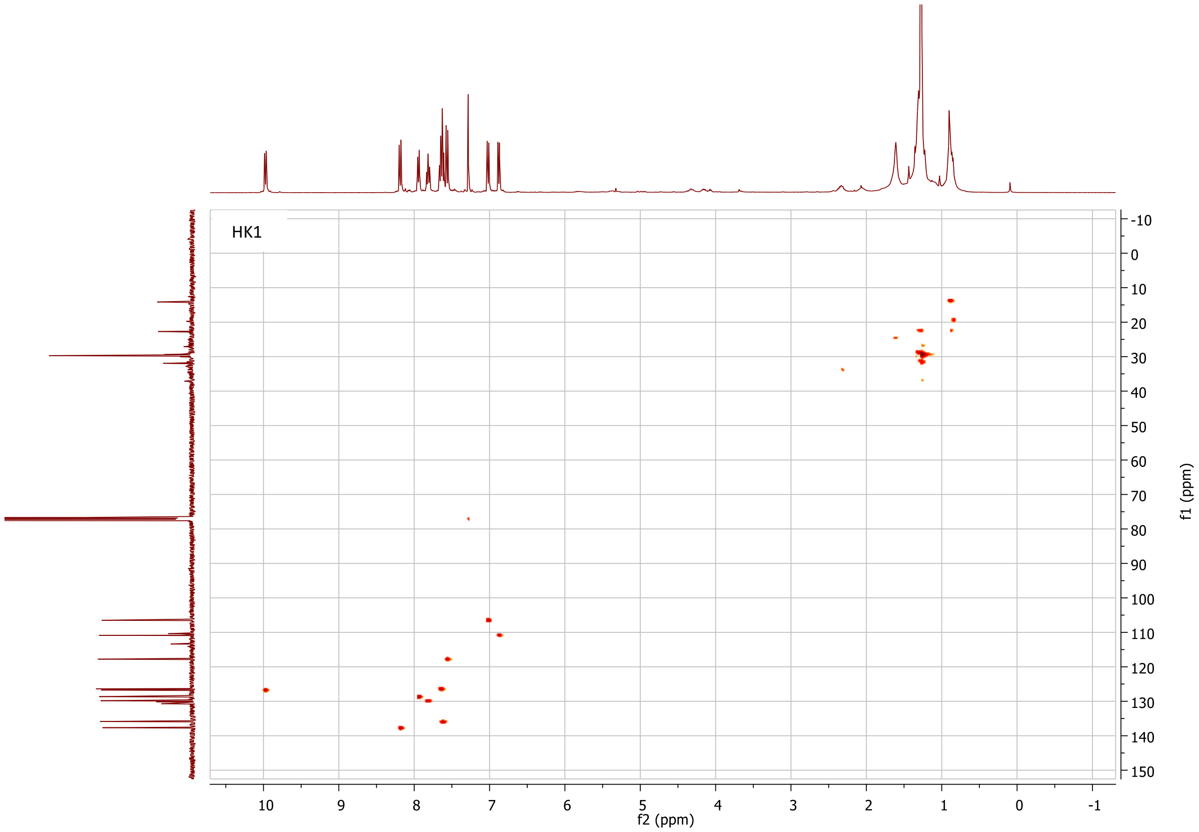

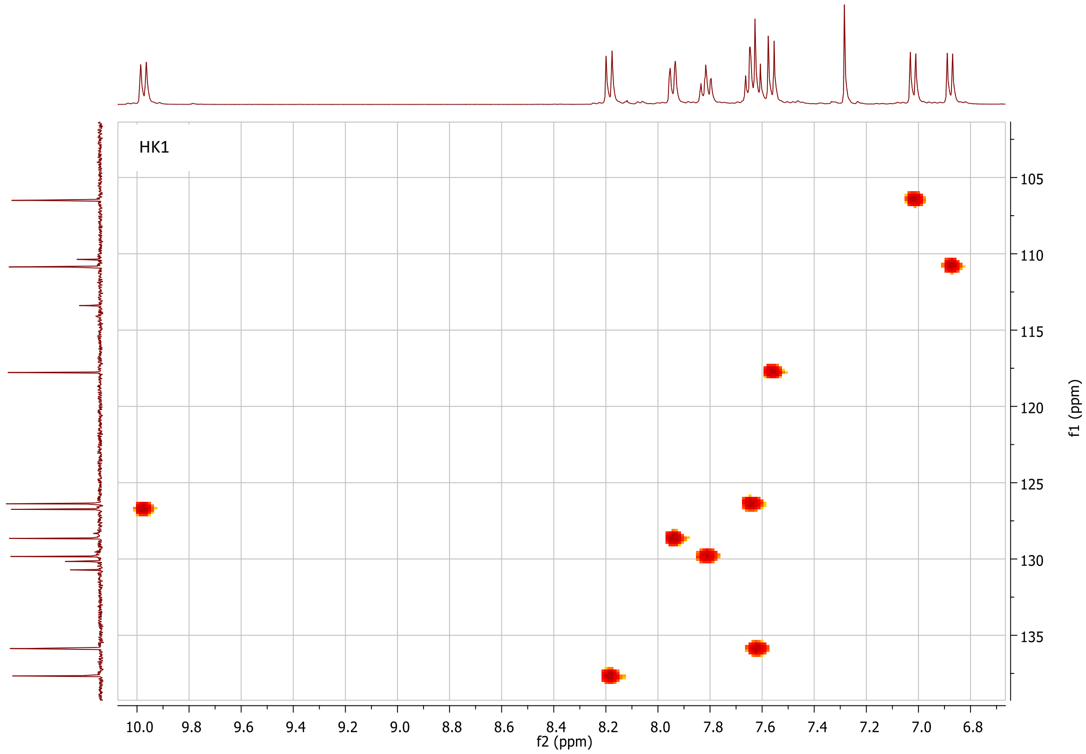

Figure S8

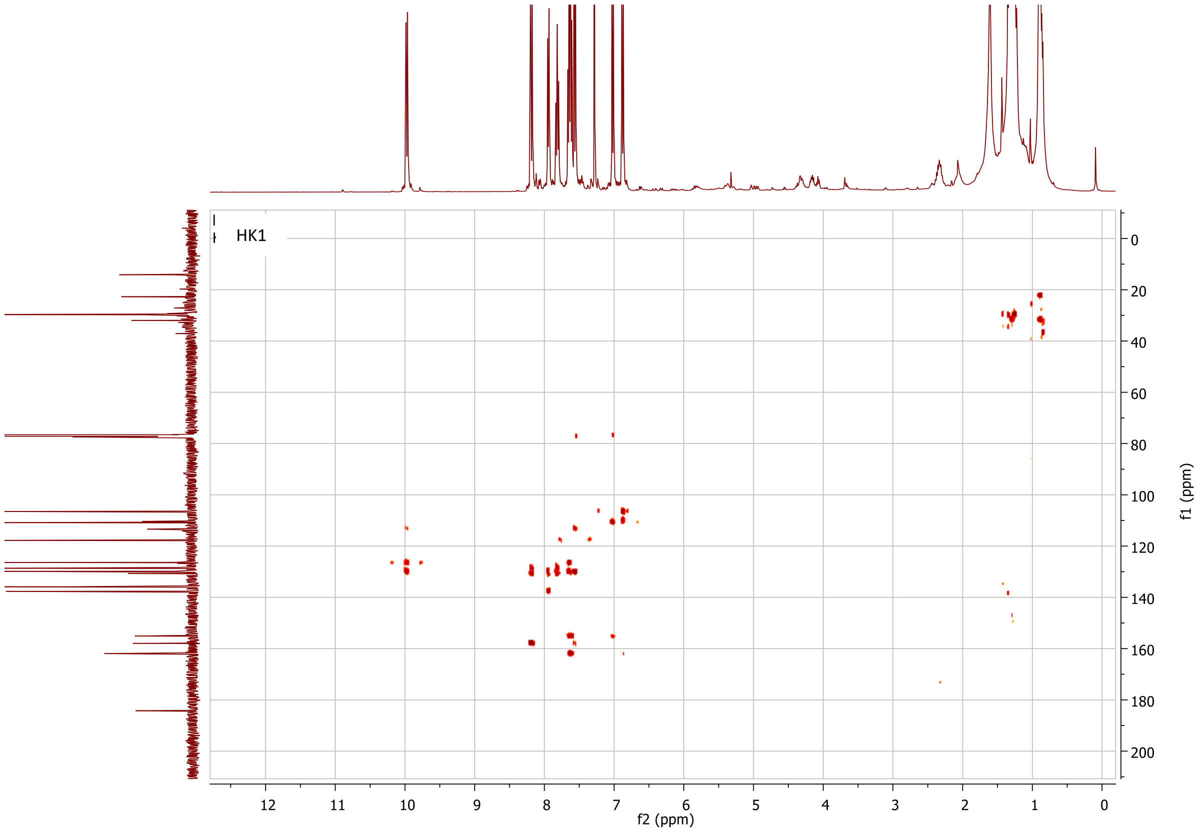

HK1
0
20
40
60
80
100
120
140
160
180
200
f1 (ppm)
12
11
10
9
8
7
6
5
4
3
2
1
0
f2 (ppm)

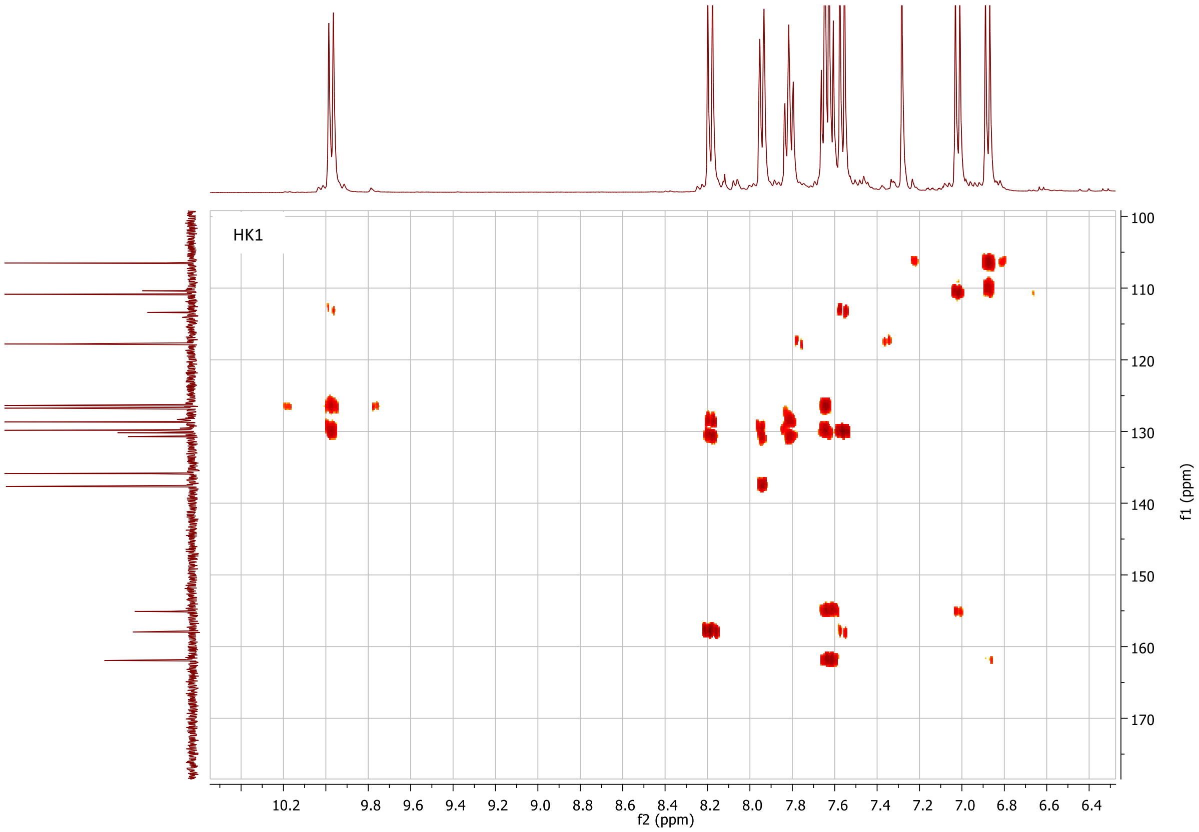

HK1
100
110
120
130
140
150
160
170
f1 (ppm)
10.2
9.8
9.6
9.4
9.2
9.0
8.8
8.6
8.4
8.2
8.0
7.8
7.6
7.4
7.2
7.0
6.8
6.6
6.4
f2 (ppm)

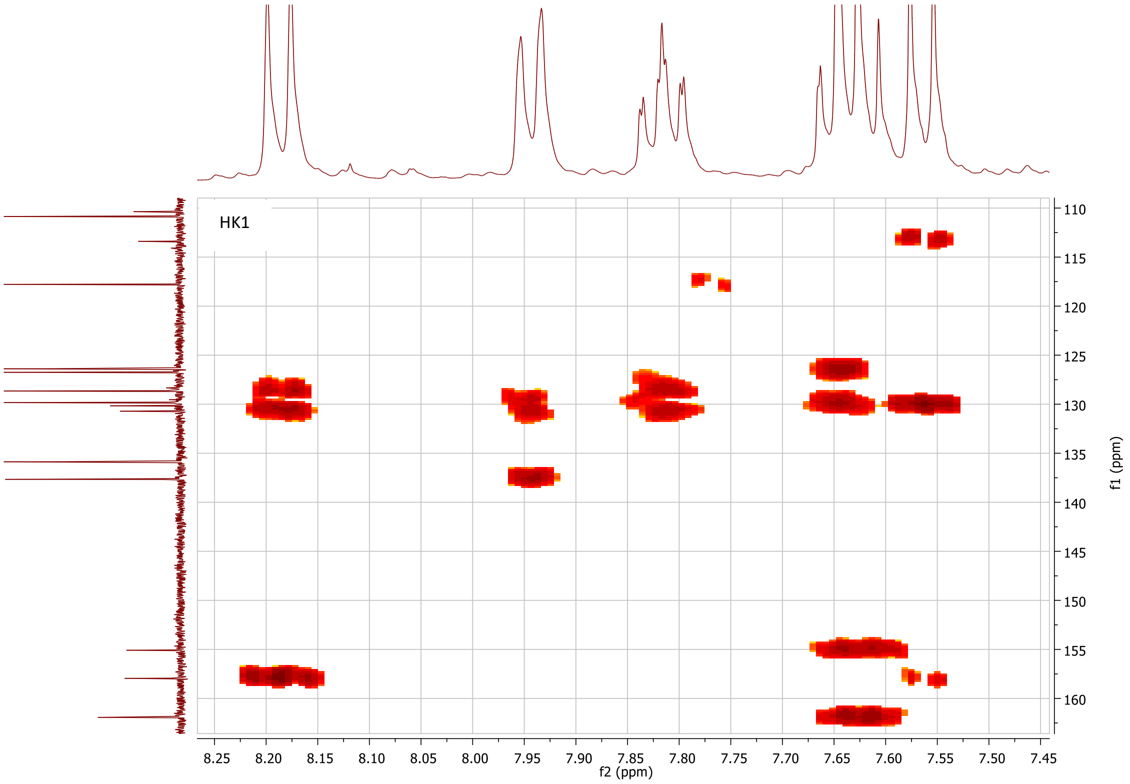
HK1
f2 (ppm)
f1 (ppm)

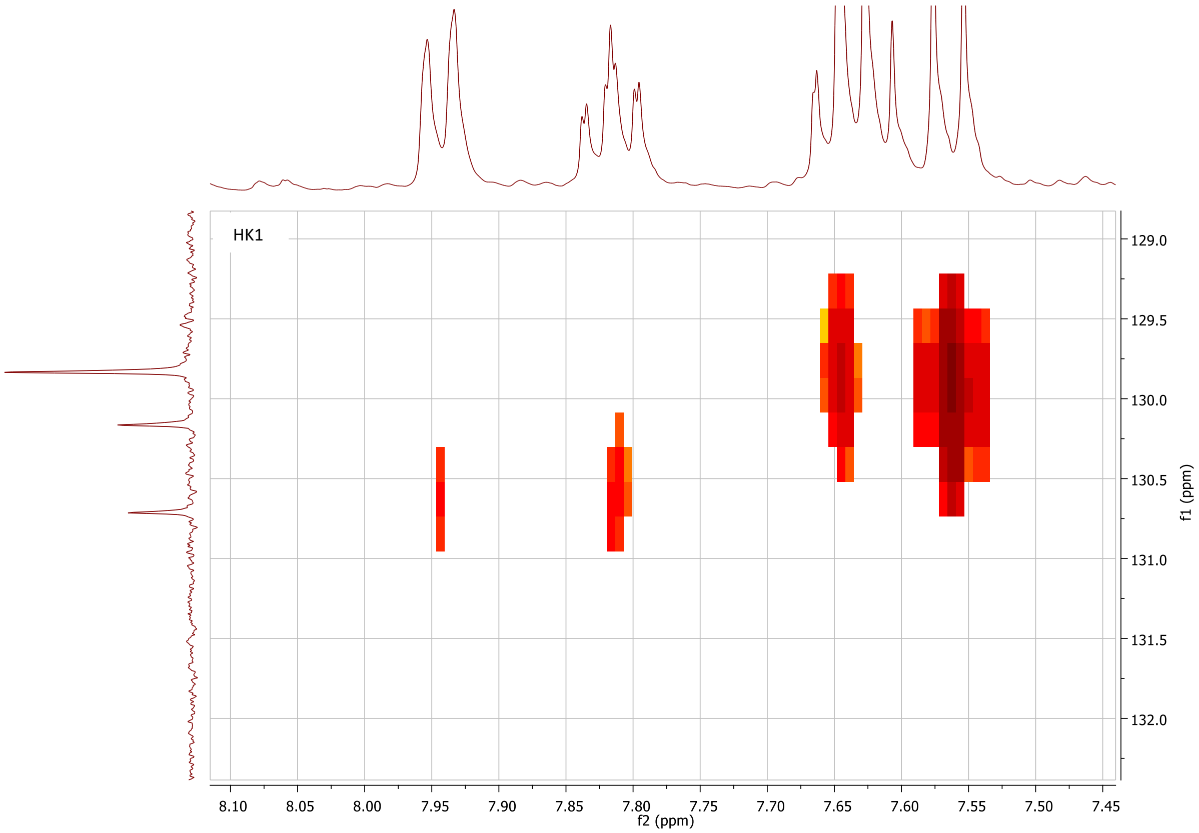
HK1
f2 (ppm)
f1 (ppm)

Figure S9

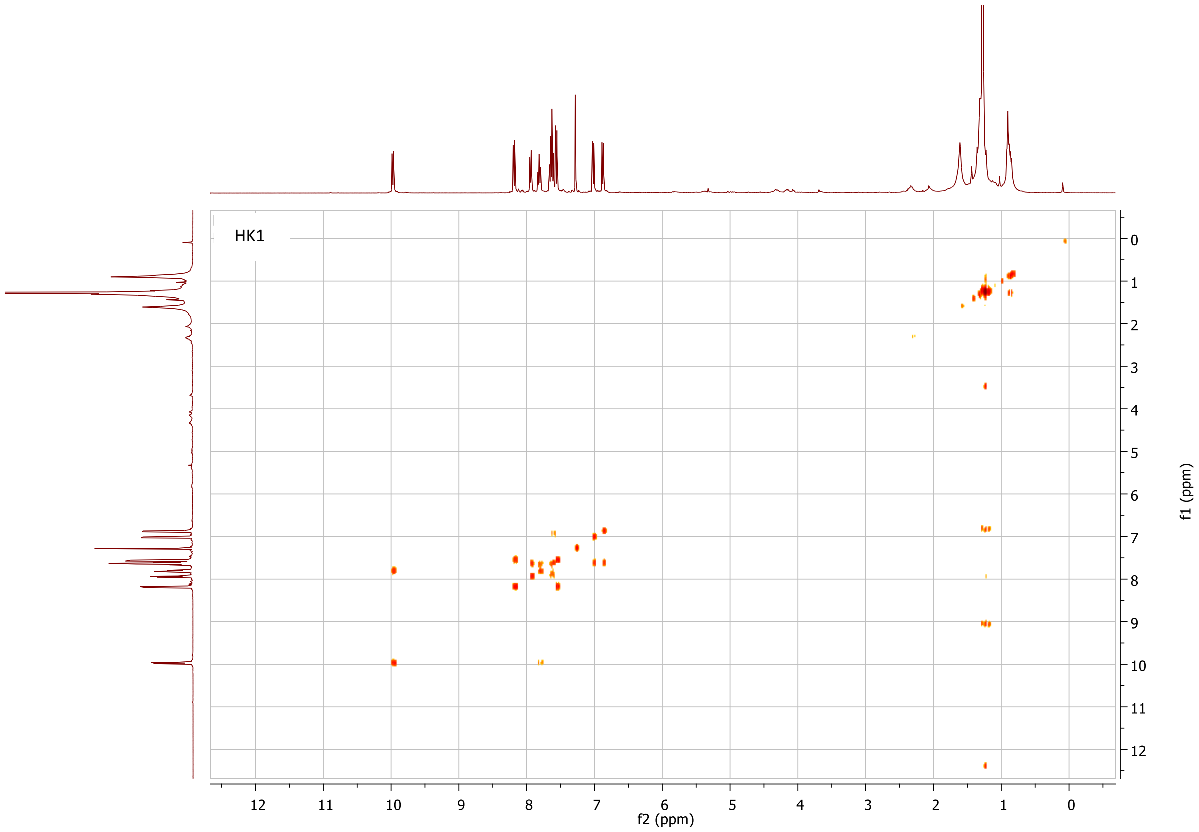

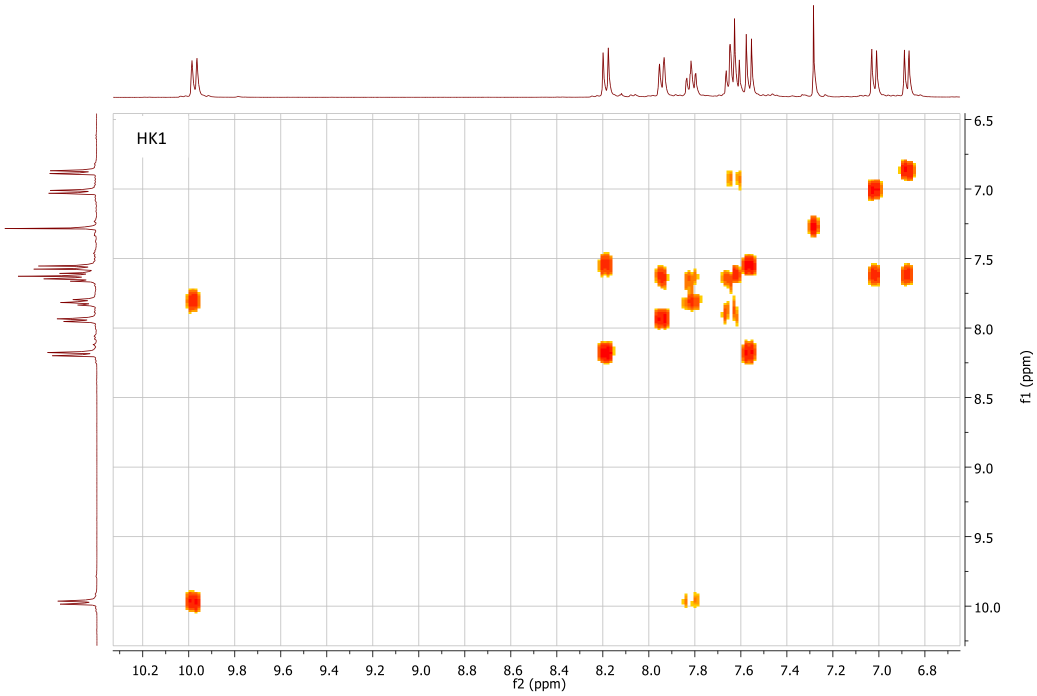

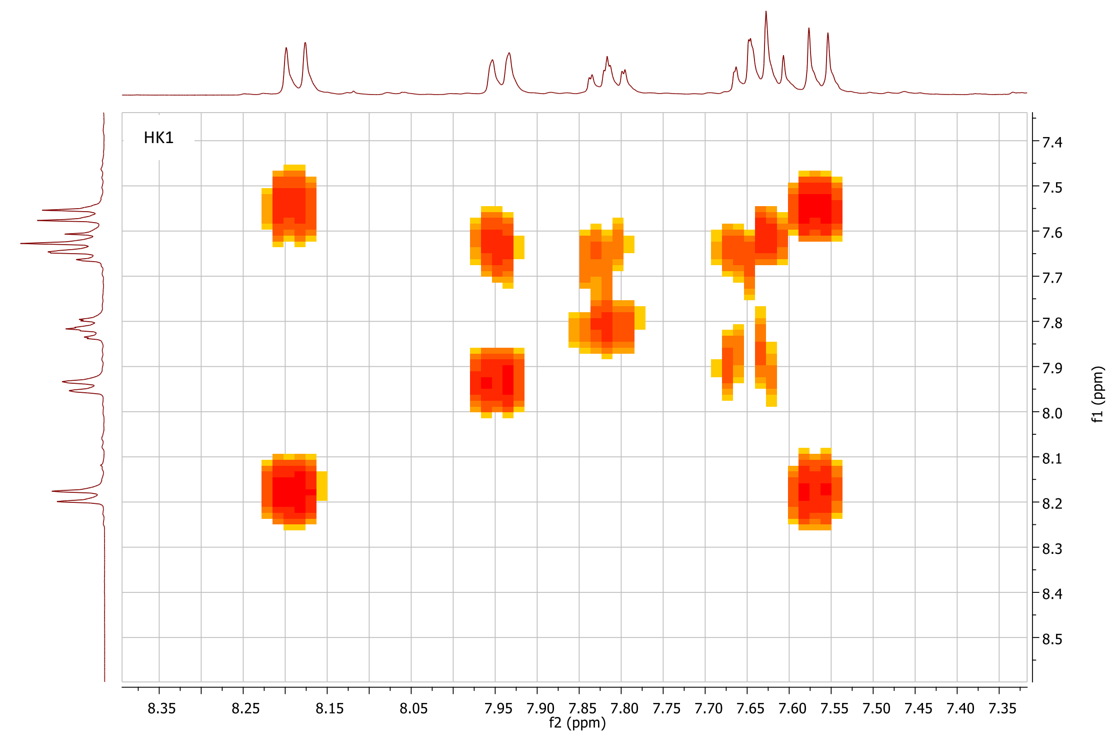
HK1
8.35 8.25 8.15 8.05 7.95 7.90 7.85 7.80 7.75 7.70 7.65 7.60 7.55 7.50 7.45 7.40 7.35
f2 (ppm)
7.4 7.5 7.6 7.7 7.8 7.9 8.0 8.1 8.2 8.3 8.4 8.5
f1 (ppm)

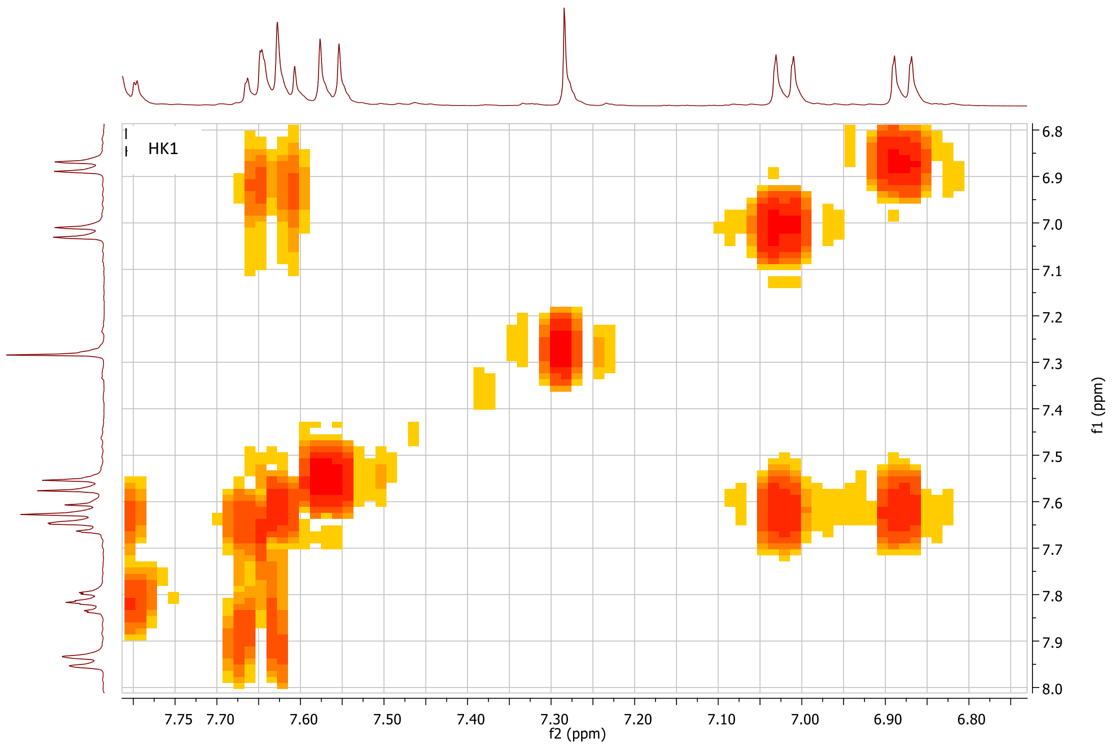
HK1
7.75 7.70 7.60 7.50 7.40 7.30 7.20 7.10 7.00 6.90 6.80
f2 (ppm)
6.8 6.9 7.0 7.1 7.2 7.3 7.4 7.5 7.6 7.7 7.8 7.9 8.0
f1 (ppm)

Compound 2 (HK2): 6-hydroxy-3-methoxy-7H-benzo[de]anthracen-7-one.

Figure S10

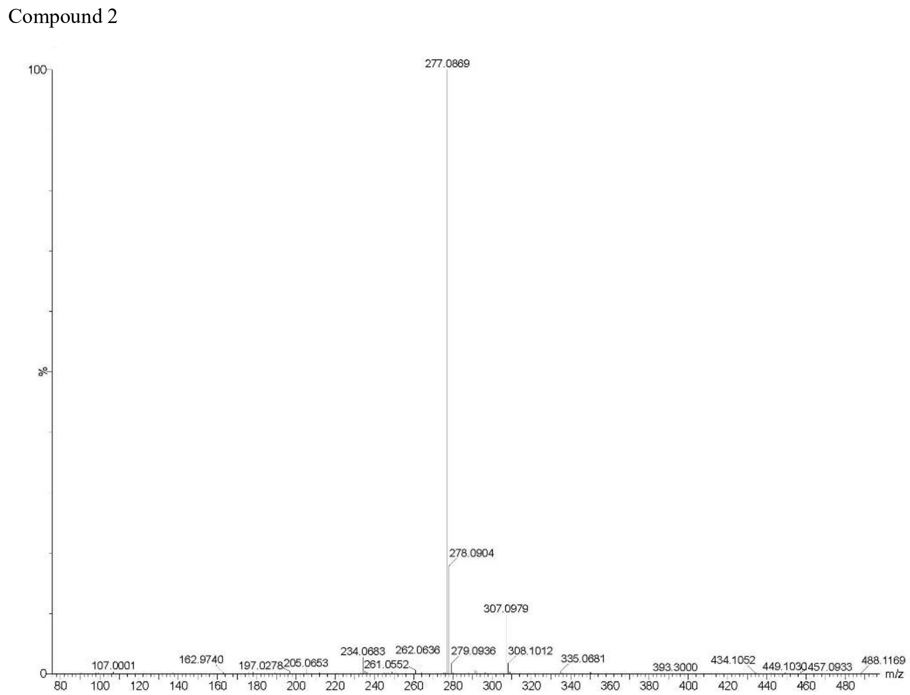

Figure S11

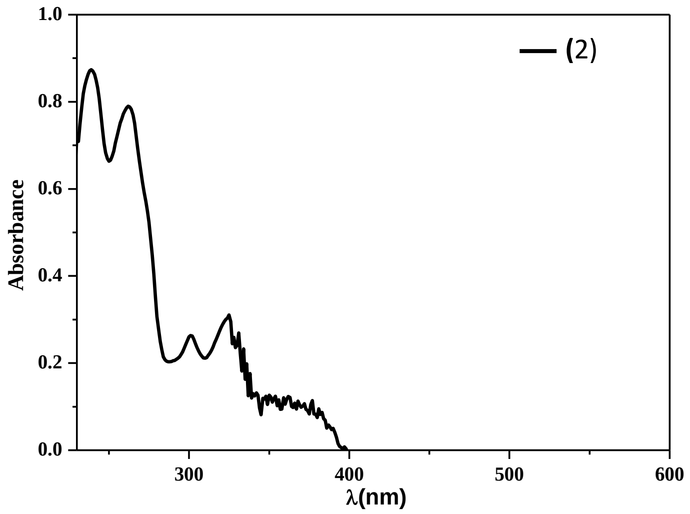

Figure S12

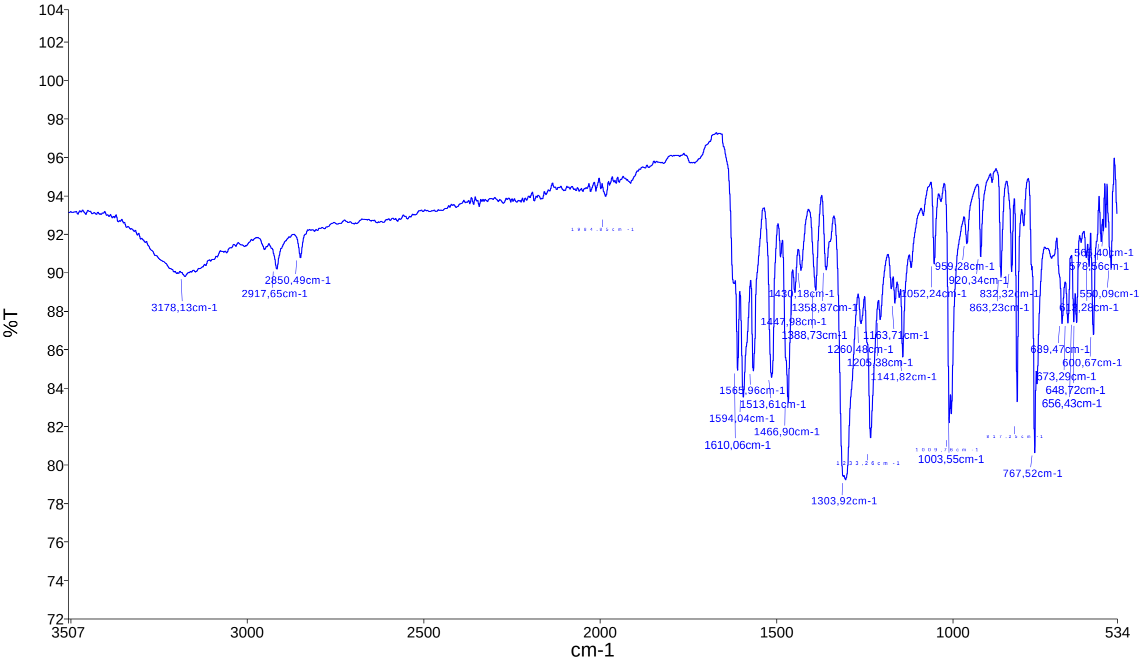
%T
cm-1
3507
3000
2500
2000
1500
1000
534
104
102
100
98
96
94
92
90
88
86
84
82
80
78
76
74
72
3178,13cm-1
2917,65cm-1
2850,49cm-1
1610,06cm-1
1594,04cm-1
1565,96cm-1
1513,61cm-1
1466,90cm-1
1447,98cm-1
1430,18cm-1
1388,73cm-1
1358,87cm-1
1303,92cm-1
1260,48cm-1
1233,26cm-1
1205,38cm-1
1163,71cm-1
1141,82cm-1
1052,24cm-1
1009,76cm-1
1003,55cm-1
959,28cm-1
920,34cm-1
863,23cm-1
832,32cm-1
817,25cm-1
767,52cm-1
689,47cm-1
673,29cm-1
656,43cm-1
648,72cm-1
600,67cm-1
578,56cm-1
550,09cm-1

Figure S13

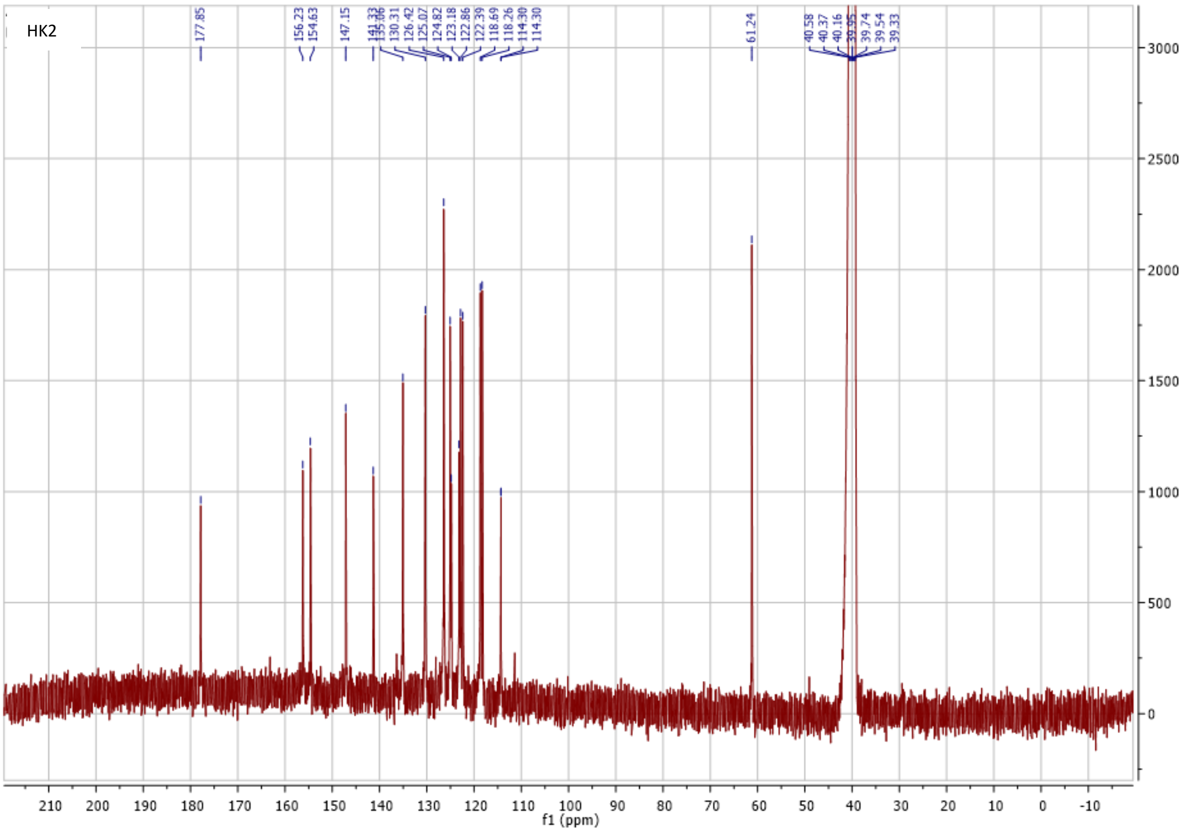
HK2
177.85
156.23
154.63
147.15
141.33
135.06
130.31
126.42
125.07
124.82
123.18
122.86
122.39
118.69
118.26
114.30
114.30
61.24
40.58
40.37
40.16
39.95
39.74
39.54
39.33
f1 (ppm)

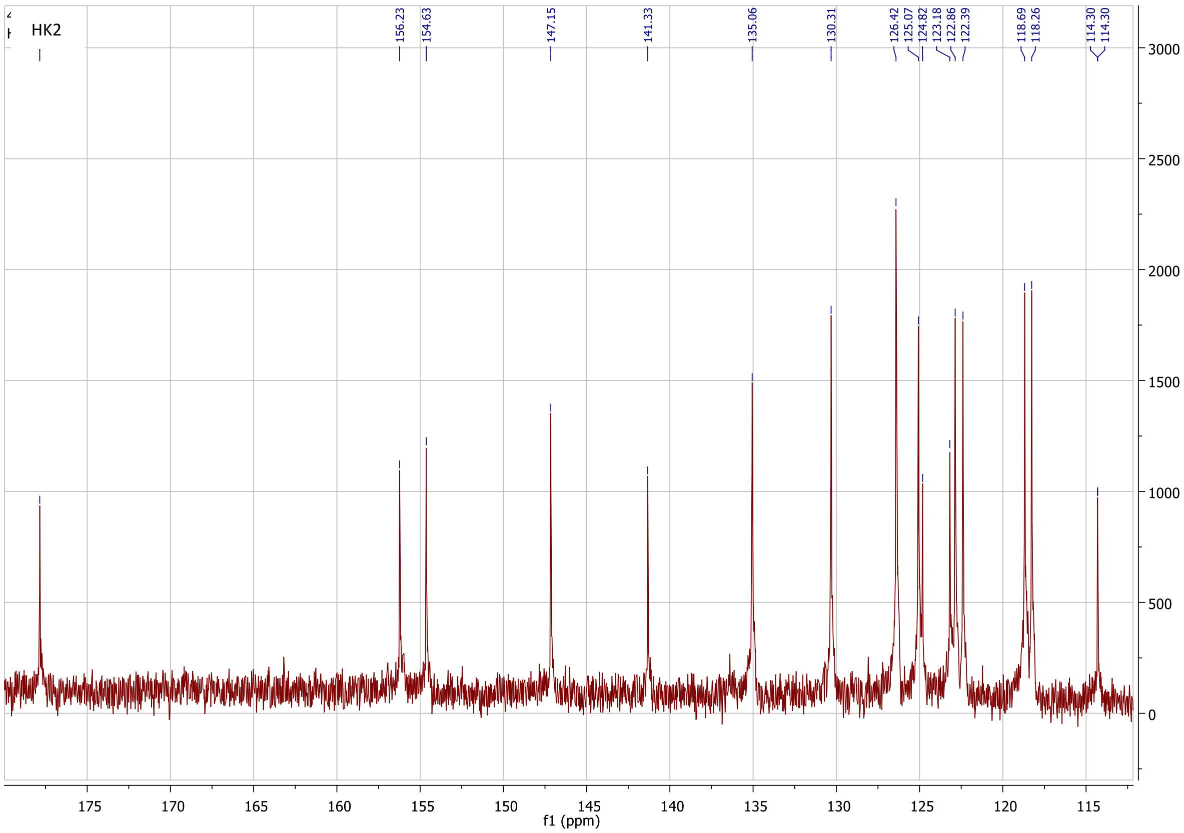
HK2
156.23
154.63
147.15
141.33
135.06
130.31
126.42
125.07
124.82
123.18
122.86
122.39
118.69
118.26
114.30
114.30
f1 (ppm)

Figure S14

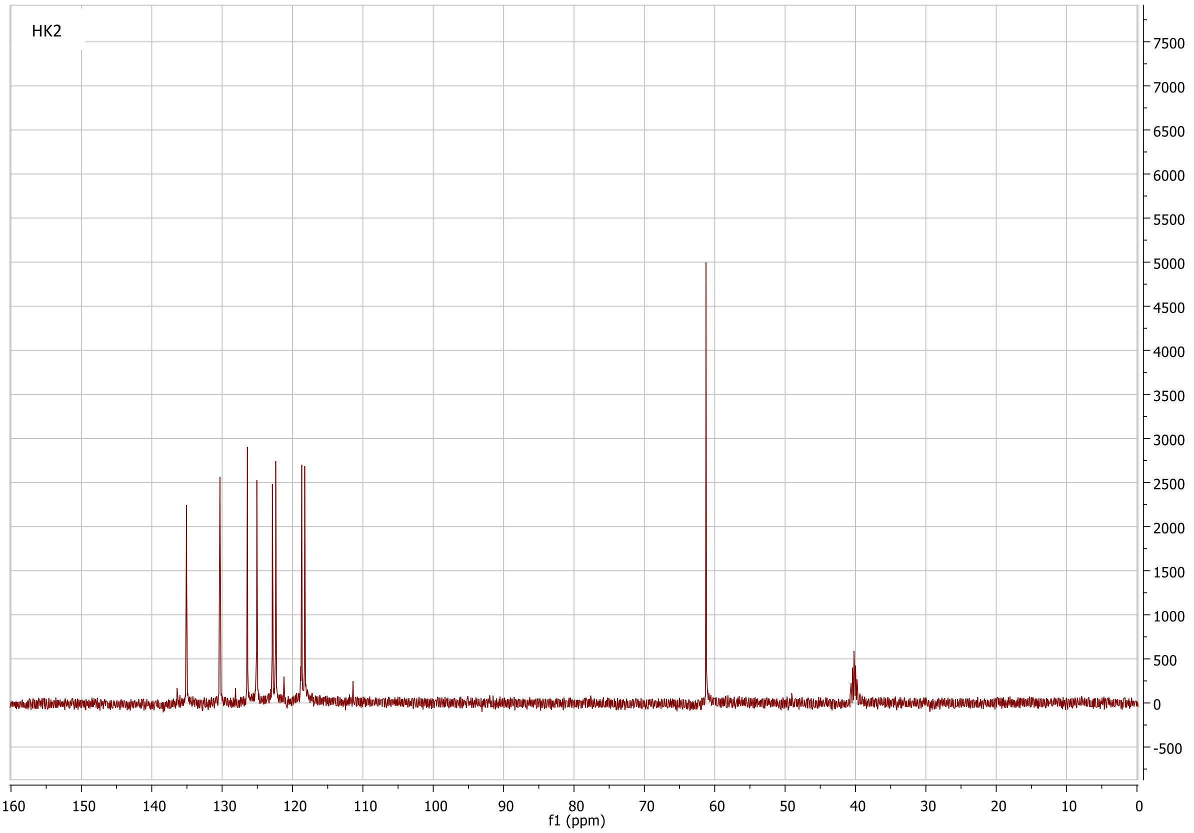

Figure S15

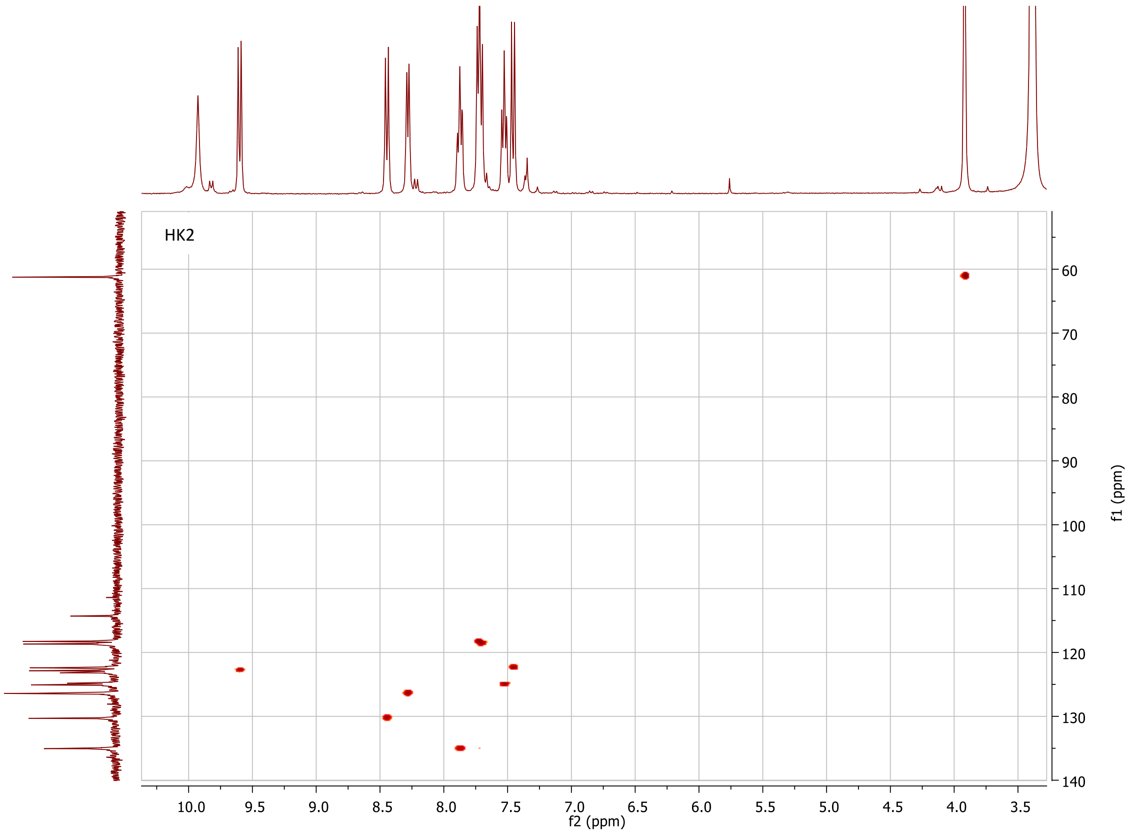

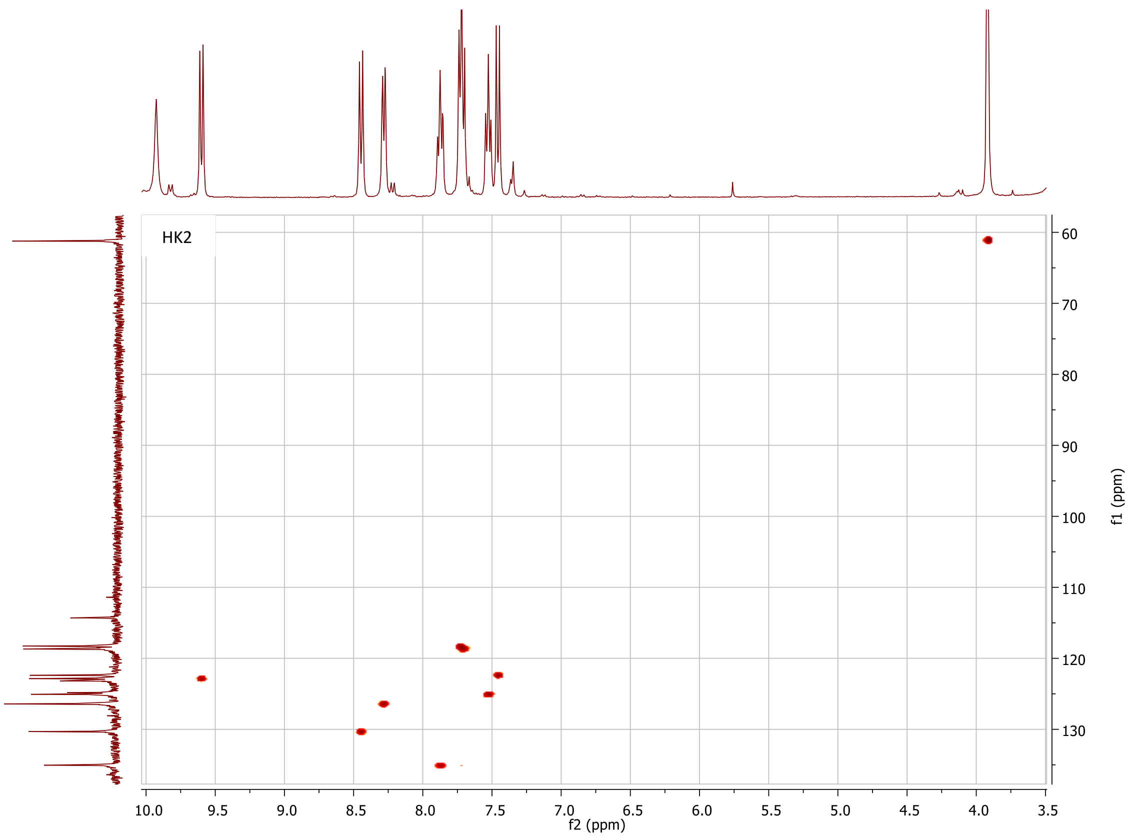
HK2
f2 (ppm)
f1 (ppm)
10.0
9.5
9.0
8.5
8.0
7.5
7.0
6.5
6.0
5.5
5.0
4.5
4.0
3.5
60
70
80
90
100
110
120
130

Figure S16

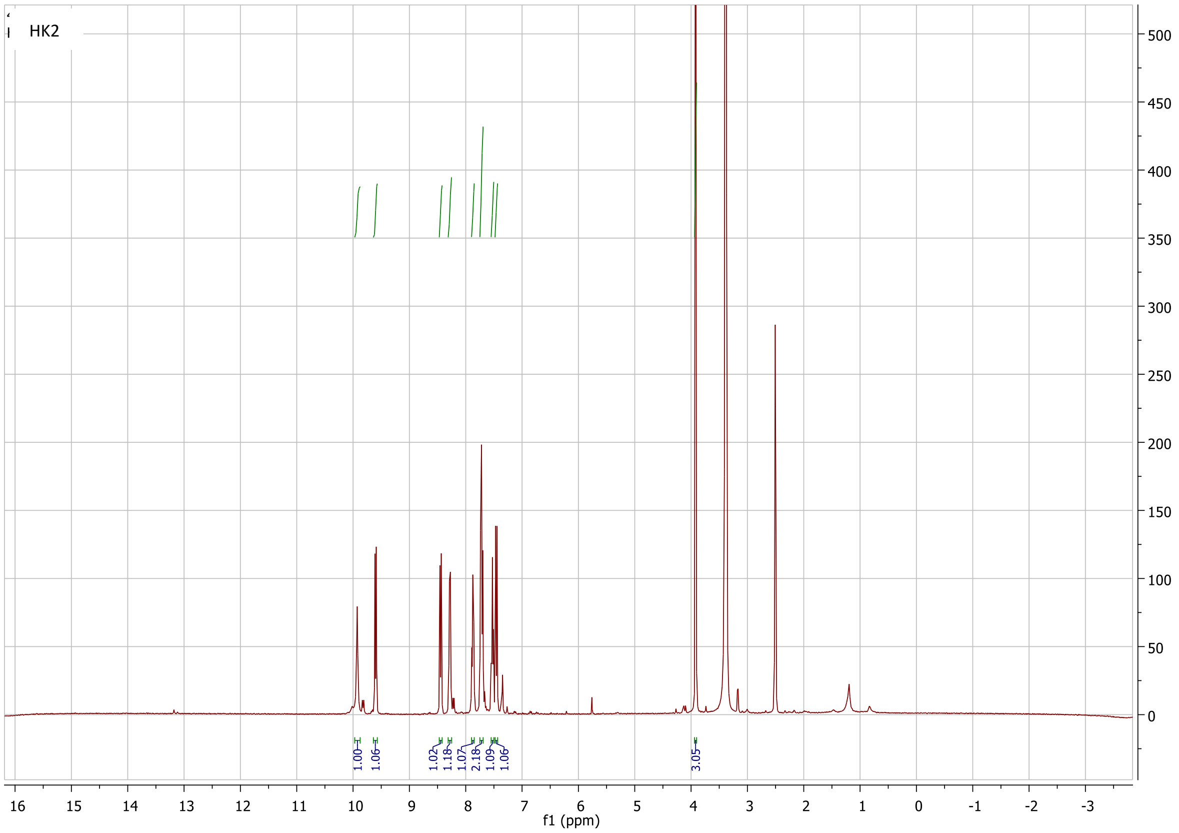

HK2
1.00
1.06
1.02
1.18
1.07
2.18
1.09
1.06
3.05
16 15 14 13 12 11 10 9 8 7 6 5 4 3 2 1 0 -1 -2 -3
f1 (ppm)
500 450 400 350 300 250 200 150 100 50 0

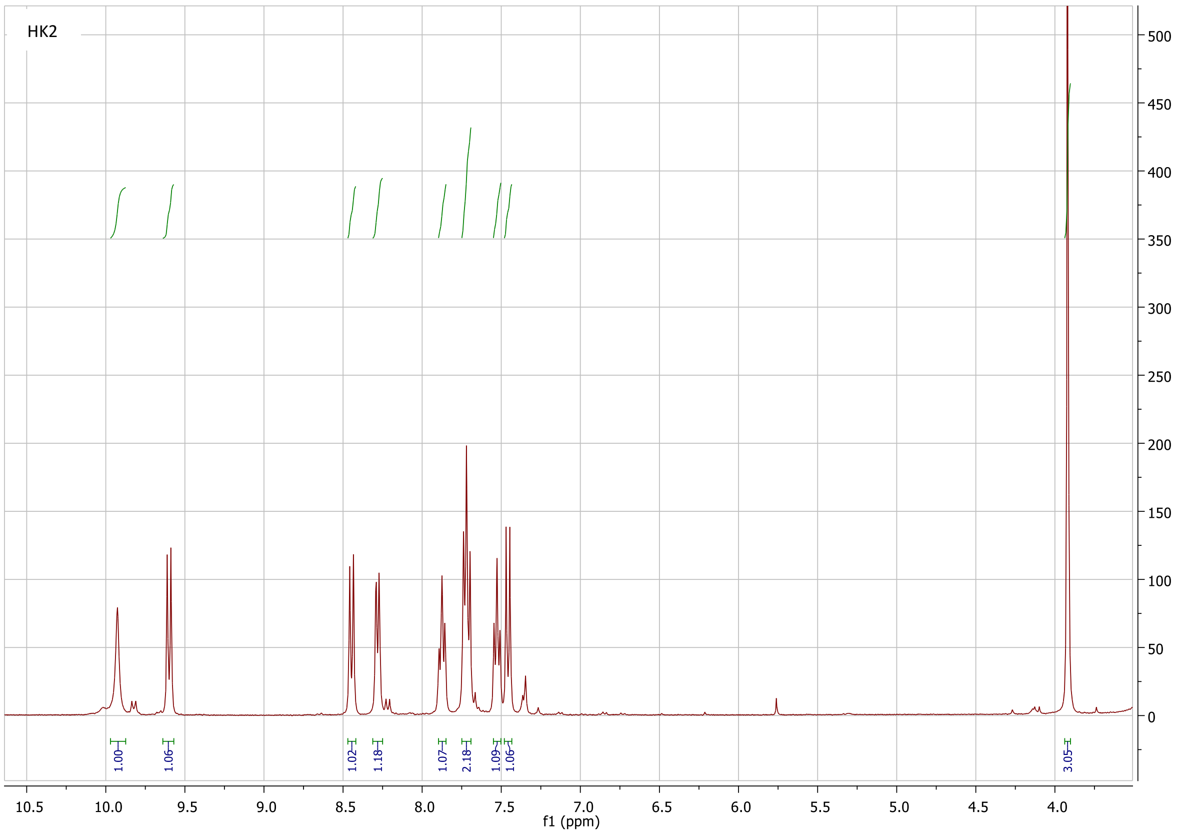

HK2
1.00
1.06
1.02
1.18
1.07
2.18
1.09
1.06
3.05
10.5 10.0 9.5 9.0 8.5 8.0 7.5 7.0 6.5 6.0 5.5 5.0 4.5 4.0
f1 (ppm)
500 450 400 350 300 250 200 150 100 50 0

Figure S17

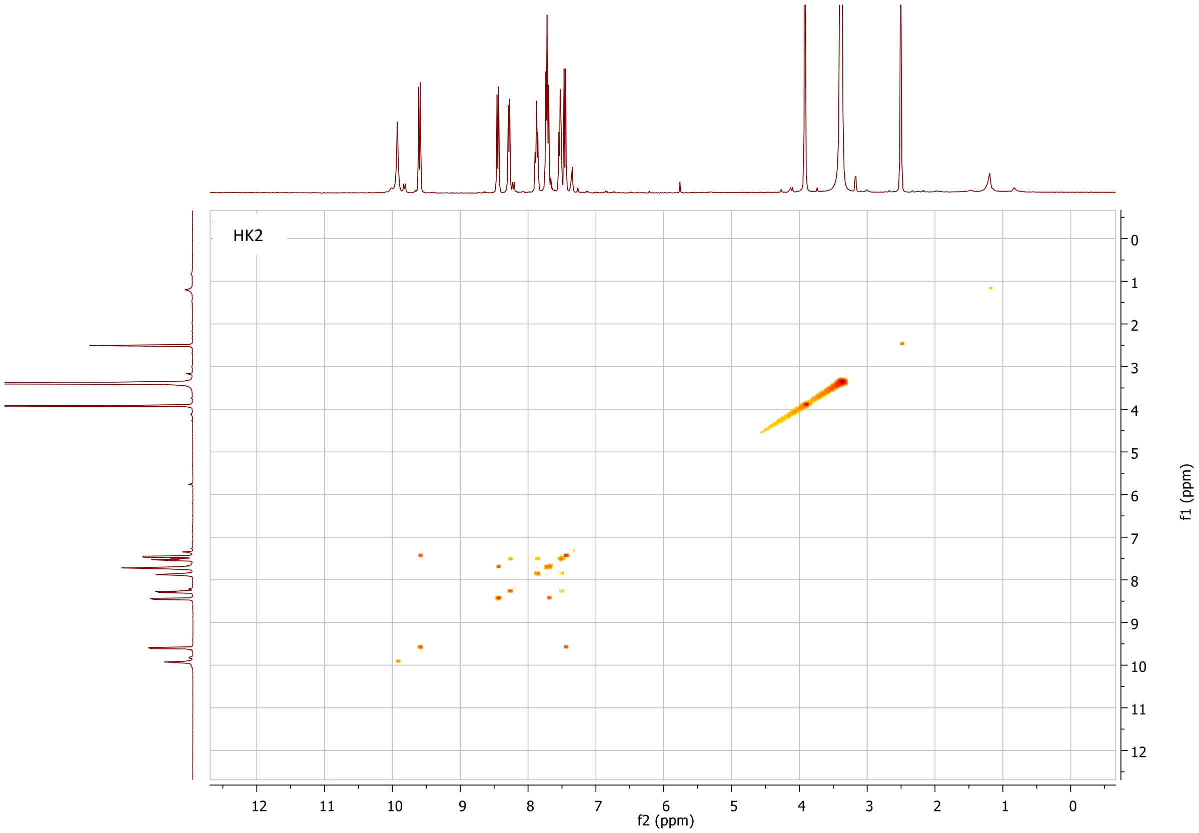
HK2
0
1
2
3
4
5
6
7
8
9
10
11
12
f1 (ppm)
12
11
10
9
8
7
6
5
4
3
2
1
0
f2 (ppm)

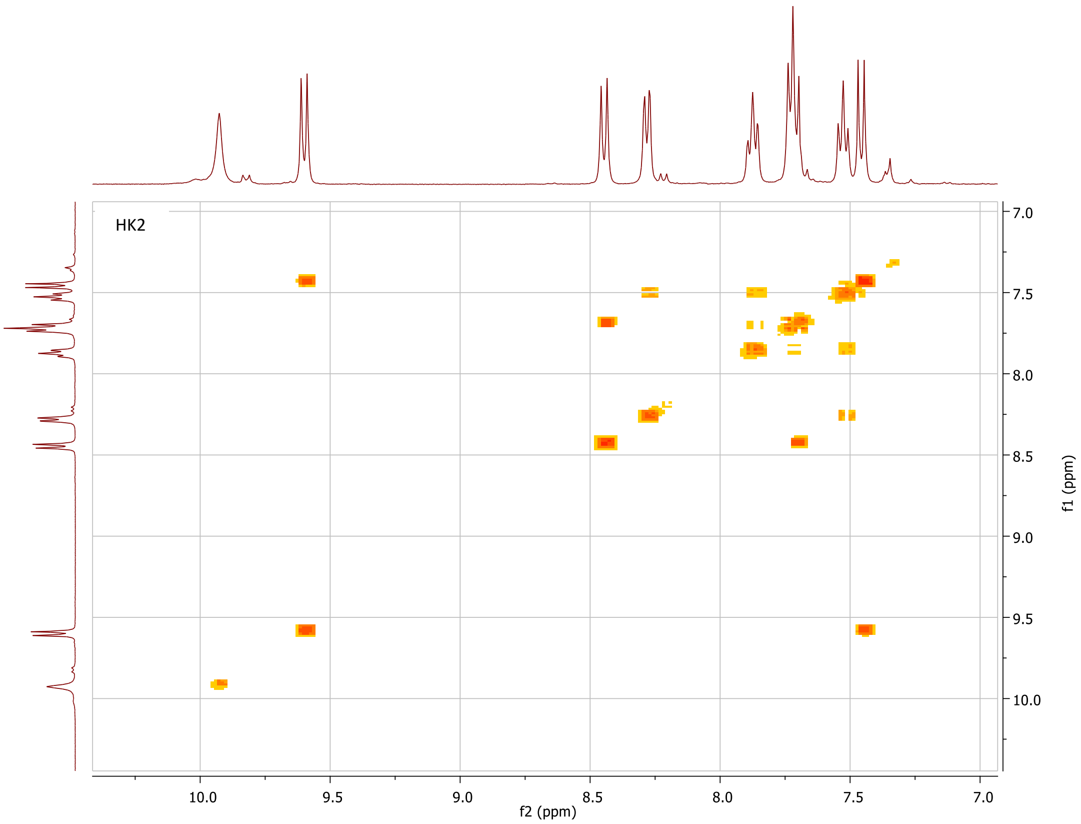
HK2
7.0
7.5
8.0
8.5
9.0
9.5
10.0
f1 (ppm)
10.0
9.5
9.0
8.5
8.0
7.5
7.0
f2 (ppm)

Figure S18

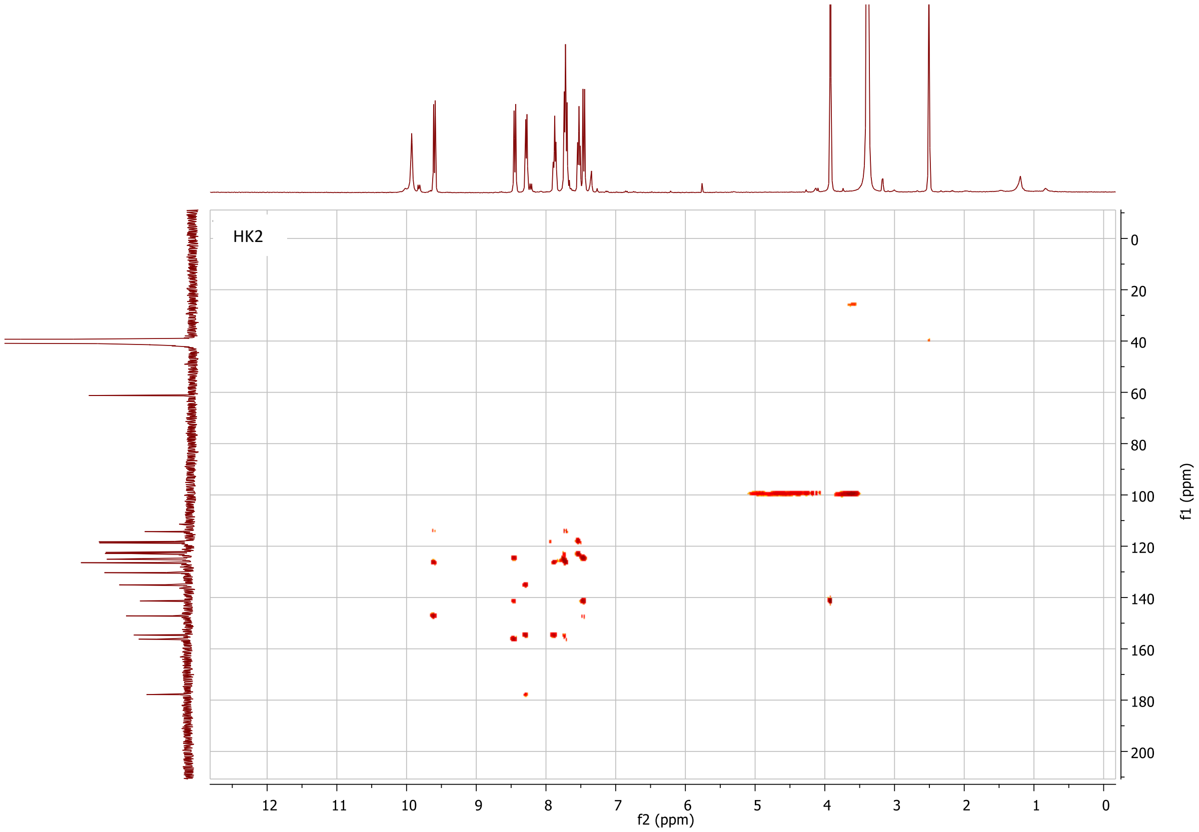
HK2
12
11
10
9
8
7
6
5
4
3
2
1
0
f2 (ppm)
0
20
40
60
80
100
120
140
160
180
200
f1 (ppm)

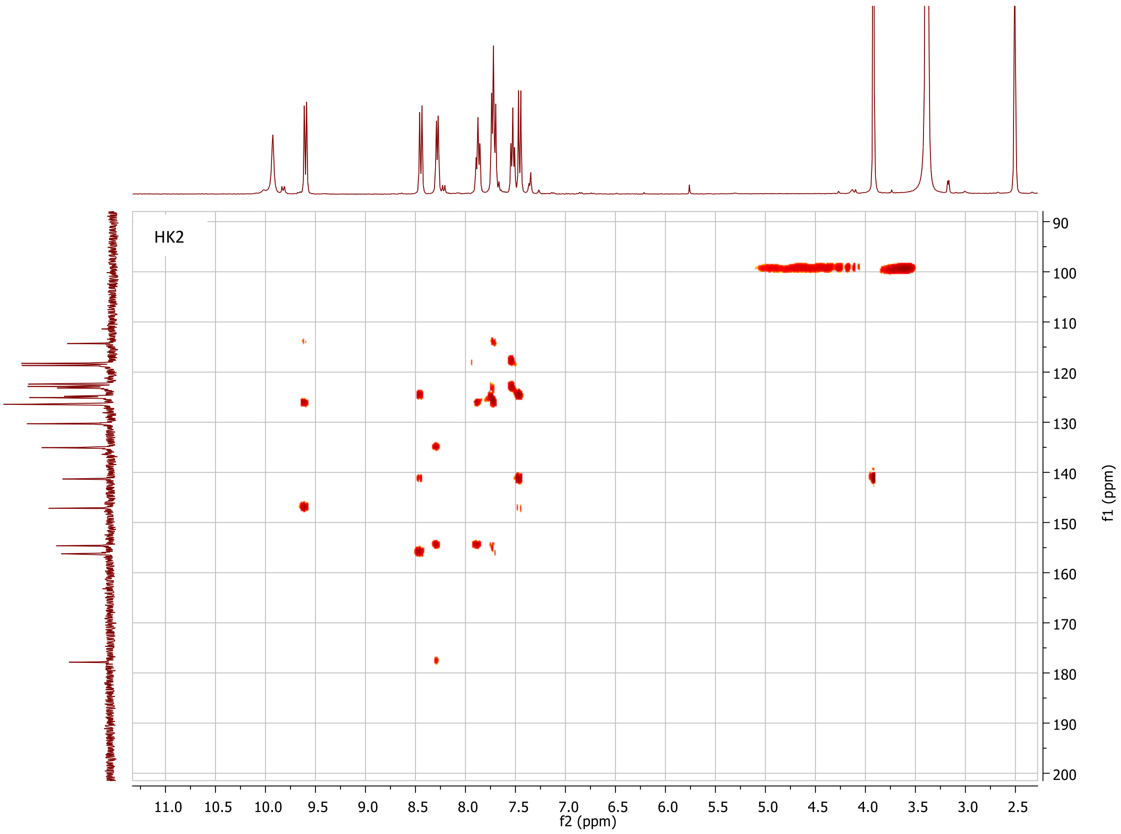
HK2
11.0
10.5
10.0
9.5
9.0
8.5
8.0
7.5
7.0
6.5
6.0
5.5
5.0
4.5
4.0
3.5
3.0
2.5
f2 (ppm)
90
100
110
120
130
140
150
160
170
180
190
200
f1 (ppm)

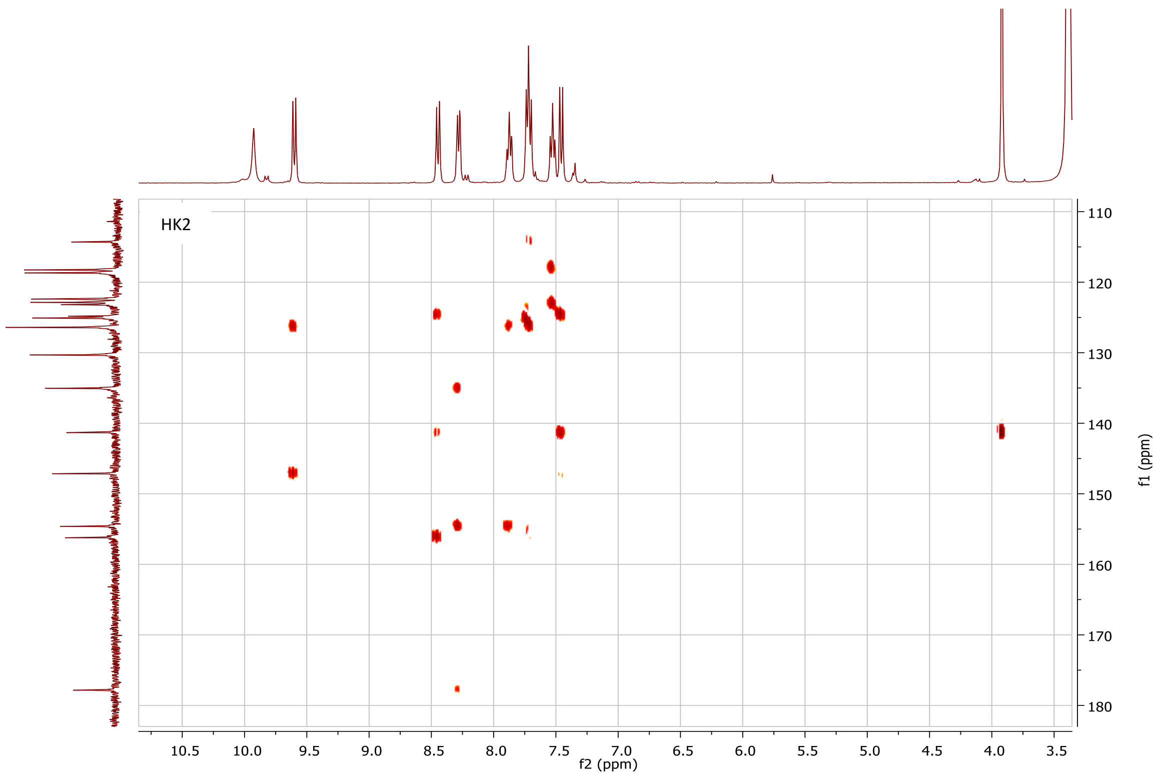
HK2
f2 (ppm)
f1 (ppm)
10.5
10.0
9.5
9.0
8.5
8.0
7.5
7.0
6.5
6.0
5.5
5.0
4.5
4.0
3.5
110
120
130
140
150
160
170
180
